# Comprehensive multiplexed immune profiling of the ductal carcinoma in situ immune microenvironment regarding subsequent ipsilateral invasive breast cancer risk

**DOI:** 10.1038/s41416-022-01888-2

**Published:** 2022-06-29

**Authors:** Mathilde M. Almekinders, Tycho Bismeijer, Tapsi Kumar, Fei Yang, Bram Thijssen, Rianne van der Linden, Charlotte van Rooijen, Shiva Vonk, Baohua Sun, Edwin R. Parra Cuentas, Ignacio I. Wistuba, Savitri Krishnamurthy, Lindy L. Visser, Iris M. Seignette, Ingrid Hofland, Joyce Sanders, Annegien Broeks, Jason K. Love, Brian Menegaz, Lodewyk Wessels, Alastair M. Thompson, Karin E. de Visser, Erik Hooijberg, Esther Lips, Andrew Futreal, Jelle Wesseling, Grand Challenge PRECISION Consortium, Esther Lips, Jelle Wesseling, Lodewyk Wessels, Andrew Futreal, Alastair M. Thompson

**Affiliations:** 1 Division of Molecular Pathology, Netherlands Cancer Institute, Amsterdam, The Netherlands; 2 Department of Pathology, Antoni van Leeuwenhoek Hospital, Amsterdam, The Netherlands; 3 Department of Pathology, Leiden University Medical Center, Leiden, The Netherlands; 4 Division of Molecular Carcinogenesis, Netherlands Cancer Institute, Amsterdam, The Netherlands; 5 Department of Genomic Medicine, MD Anderson Cancer Center, Houston, TX USA; 6 Department of Genetics, MD Anderson Cancer Center, Houston, TX USA; 7 MD Anderson Cancer Center UT Health Graduate School of Biomedical Sciences, Houston, TX USA; 8 Department of Translational Molecular Pathology, MD Anderson Cancer Center, Houston, TX USA; 9 Core Facility Molecular Pathology and Biobanking, Netherlands Cancer Institute, Antoni van Leeuwenhoek Hospital, Amsterdam, The Netherlands; 10 Department of Pathology, MD Anderson Cancer Center, Houston, TX USA; 11 Breast Surgical Oncology, MD Anderson Cancer Center, Houston, TX USA; 12 Department of Surgery, Baylor College of Medicine, Houston, TX USA; 13 Oncode Institute, Utrecht, The Netherlands; 14 Division of Surgical Oncology, Baylor College of Medicine, Houston, TX USA; 15 Division of Tumour Biology & Immunology, Netherlands Cancer Institute, Amsterdam, The Netherlands; 16 Department of Immunology, Leiden University Medical Center, Leiden, The Netherlands; 17 Division of Molecular Pathology, Netherlands Cancer Institute, Amsterdam, The Netherlands; 18 Department of Pathology, Antoni van Leeuwenhoek Hospital, Amsterdam, The Netherlands; 19 Department of Pathology, Leiden University Medical Center, Leiden, The Netherlands; 20 Division of Molecular Carcinogenesis, Netherlands Cancer Institute, Amsterdam, The Netherlands; 21 Oncode Institute, Utrecht, The Netherlands; 22 Department of Genomic Medicine, MD Anderson Cancer Center, Houston, TX USA; 23 Division of Surgical Oncology, Baylor College of Medicine, Houston, TX USA

**Keywords:** Prognostic markers, Imaging the immune system, Breast cancer, Cancer microenvironment, Translational research

## Abstract

**Background:**

Ductal carcinoma in situ (DCIS) is treated to prevent subsequent ipsilateral invasive breast cancer (iIBC). However, many DCIS lesions will never become invasive. To prevent overtreatment, we need to distinguish harmless from potentially hazardous DCIS. We investigated whether the immune microenvironment (IME) in DCIS correlates with transition to iIBC.

**Methods:**

Patients were derived from a Dutch population-based cohort of 10,090 women with pure DCIS with a median follow-up time of 12 years. Density, composition and proximity to the closest DCIS cell of CD20^+^ B-cells, CD3^+^CD8^+^ T-cells, CD3^+^CD8^−^ T-cells, CD3^+^FOXP3^+^ regulatory T-cells, CD68^+^ cells, and CD8^+^Ki67^+^ T-cells was assessed with multiplex immunofluorescence (mIF) with digital whole-slide analysis and compared between primary DCIS lesions of 77 women with subsequent iIBC (cases) and 64 without (controls).

**Results:**

Higher stromal density of analysed immune cell subsets was significantly associated with higher grade, ER negativity, HER-2 positivity, Ki67 ≥ 14%, periductal fibrosis and comedonecrosis (*P* < 0.05). Density, composition and proximity to the closest DCIS cell of all analysed immune cell subsets did not differ between cases and controls.

**Conclusion:**

IME features analysed by mIF in 141 patients from a well-annotated cohort of pure DCIS with long-term follow-up are no predictors of subsequent iIBC, but do correlate with other factors (grade, ER, HER2 status, Ki-67) known to be associated with invasive recurrences.

## Graphical abstract

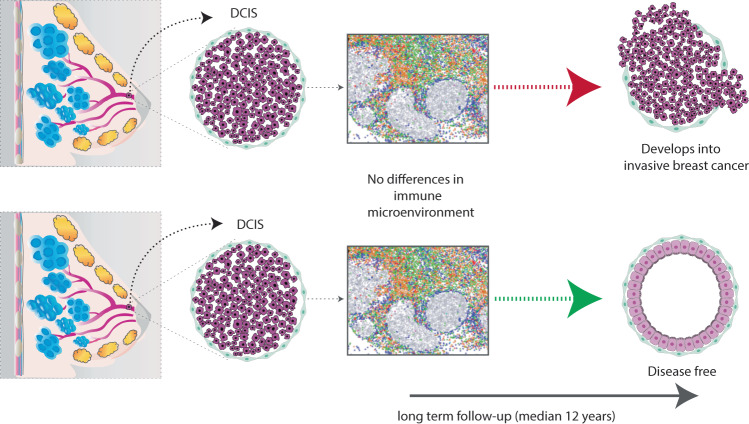

## Introduction

Ductal carcinoma in situ (DCIS) is a non-obligate precursor to invasive breast cancer (IBC). DCIS incidence has dramatically increased in countries where population-based mammographic screening was introduced [1–4]. The current treatment for DCIS involves surgery and radiotherapy, sometimes followed by endocrine therapy. Yet, the majority of DCIS lesions will never progress to IBC, suggesting overdiagnosis and overtreatment [5–8]. Therefore, prognostic markers that can distinguish harmless from potentially hazardous DCIS are urgently needed.

As yet, it is unclear what causes DCIS to progress to ipsilateral invasive breast cancer (iIBC), but the immune-microenvironment (IME) might play a crucial role here. Immune cells might either drive DCIS lesions to become invasive, facilitate immune escape, prevent invasion or be indicators for tumour aggressiveness.

Several studies have investigated whether characteristics of the IME in DCIS are associated with outcome. In a patient series of Pinder et al., presence of chronic inflammation in DCIS was associated with local recurrence [9]. Pruneri et al. found no association between percentage of stromal tumour infiltrating lymphocytes (TILs) and a second breast event after DCIS [10]. Higher TIL density was associated with a shorter (ipsilateral) recurrence-free survival (RFS) in a DCIS series by Thike et al. [11]. and Darvishian et al. [12]. In a subgroup of DCIS patients treated with breast conserving surgery (BCS) without radiotherapy, presence of >5% TILs correlated with reduced risk for second breast event[13].

The search for markers and features of the IME related to recurrence risk after primary DCIS further yielded several candidates, including FOXP3^+^ TILs, PD-L1^+^ immune cells, FoxP3/CD8 and FoxP3/CD4 ratio [14], PDL-1 expression in DCIS cells [15], CD8^+^HLADR^−^ T-cells, CD8^+^HLADR^+^ T-cells, and CD115^+^ cells [16], CD68^+^ and CD163^+^ macrophages [17], and touching TILs (TILs touching or closely approximating the DCIS ducts’ basement membrane) [18]. Immune parameters that were associated with poorer RFS for subsequent iIBC specifically, included higher CD4^+^ T-cell density, high CD4^+^/CD8^+^ ratio [11] and high density of CD163^+^ macrophages [17]. Gil Del Alcazar et al. suggested that the frequency of activated CD8^+^ T-cells may predict which DCIS lesions will progress to iIBC [19]. Despite all efforts, to date no robust and reproducible prognostic features of the IME have entered clinical practice yet. The results of many studies may have been impacted by a small sample size. Most studies do not distinguish between in situ and invasive recurrences or do not stratify for treatment (mastectomy, BCS with or without radiotherapy), while these factors are pivotal for outcome. Moreover, in order to investigate the prognostic value of immune cell characteristics, large, well-annotated patient cohorts with pure DCIS, long-term follow-up and uniform treatment are necessary.

The present case–control study is an investigation of immune cell subsets within the IME on whole slides in our unique and well-annotated DCIS cohort with long-term follow-up (median 12.0 years) [7, 20], comparing 77 patients with pure DCIS that developed iIBC with 64 patients that did not. The 141 DCIS patients that were included in the study were all treated with BCS alone without radiotherapy or hormonal therapy. The DCIS IME was extensively interrogated on whole slides with multiplex immunofluorescent (mIF) analysis and double-staining IHC enabling the identification of lymphocytes, CD20^+^ B-cells, CD3^+^CD8^+^ T-cells, CD3^+^CD8^−^ T-cells, CD3^+^FOXP3^+^ regulatory T-cells, CD68^+^ cells, CD8^+^Ki67^+^ T-cells and tertiary lymphoid structures (TLS) on the same tissue section, providing vast quantitative information on the density, composition and spatial distribution of immune cells. Characteristics of the DCIS IME were correlated with clinicopathological features and outcome.

## Materials and methods

### Study population and design

**Fig. 1:**
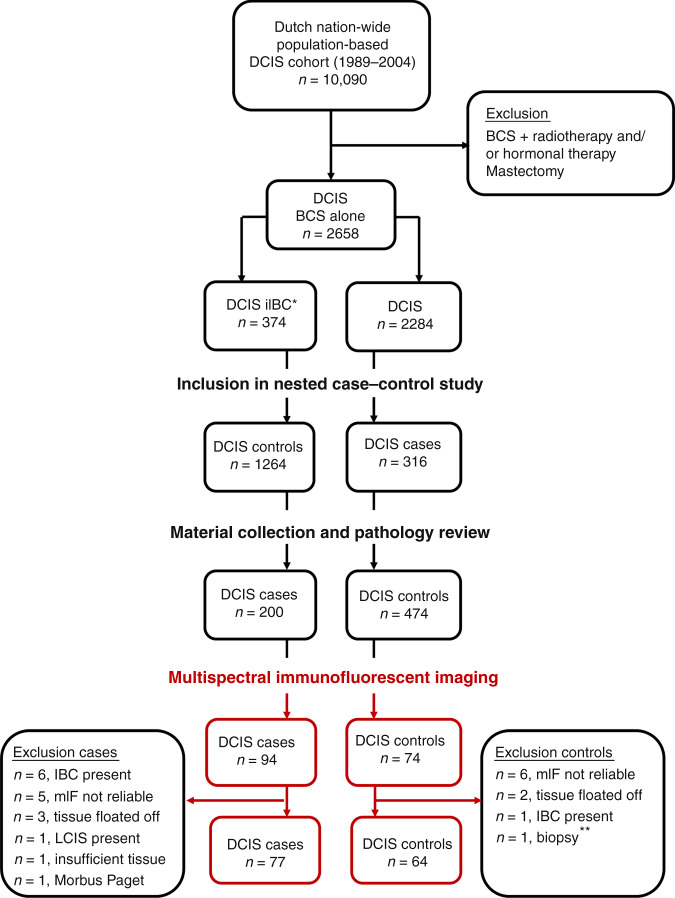
Design of the study. DCIS patients were derived from a Dutch nation-wide population-based DCIS cohort (*n* = 10,090). Within this cohort, DCIS patients were selected that were treated with breast conserving surgery (BCS) alone, of which 374 women developed a subsequent iIBC. After material collection and pathology review, 674 DCIS patients were included in a nested case–control study, in which cases represent DCIS patients that developed a subsequent iIBC, while controls did not. Lumpectomy specimens from 77 cases and 64 controls were available for multispectral immunofluorescent imaging. *Of all women diagnosed with primary DCIS and treated with BCS alone, 374 women developed a subsequent iIBC. **One control was excluded because only biopsy material was available. BCS breast conserving surgery, LCIS lobular carcinoma in situ, IBC invasive breast cancer, mIF multiplex immunofluorescence.

We included patients with pure DCIS derived from a previously described Dutch nation-wide population-based cohort of 10,090 women diagnosed and treated between 1989 and 2005, with a median follow-up of 12.0 years (IQR 9.0–15.3) [7]. Within this cohort, 2,658 women were treated with BCS alone, of whom 374 women developed an iIBC after primary DCIS diagnosis (Fig. 1) [7]. DCIS patients did not receive radiotherapy or adjuvant anti-hormonal treatment. Cases were defined as women with pure DCIS that developed subsequent iIBC at least 3 months following initial diagnosis, while women with DCIS who did not develop subsequent iIBC were designated as controls. Formalin-fixed paraffin embedded (FFPE) tissue blocks, histopathological assessment of freshly cut H&E slides and immunohistochemistry (IHC) for oestrogen receptor (ER), progesteron receptor (PR), HER2 and COX-2 were available for 185 cases and 323 controls as described previously [20]. For patients with multiple FFPE blocks, a representative whole section containing sufficient DCIS and surrounding tissue was selected. Patients were originally matched for age at DCIS diagnosis (±0–6 months). Age ranged from 33.9 to 86.7 years. Body weight was unknown.

Figure 1 shows the DCIS patients that were included in the study (77 cases and 64 controls). Since part of the DCIS patients were excluded, it was necessary to omit case–control matching for age.

### Automated multiplex immunofluorescent analysis with multispectral imaging

Multiplex IF analysis with tyramide signal amplification was performed on one representative whole tissue FFPE slide of each DCIS patient. The mIF panel consisted of CD20, CD3, CD8, FoxP3, CD68 and pancytokeratin AE1/AE3 and was stained with the automated Ventana Discovery platform. Antibody expression in the mIF panel was compared to that in single stained slides on control tonsil tissue. Staining patterns did not differ between the single- and multiplex scanned slides. Each marker was also validated with conventional IHC. Slides were scanned and analysed with the Vectra® microscope (PerkinElmer, Hopkinton, MA). Information about reagents, antibodies and detailed staining methods is provided in the supplementary methods and Supplementary Table 1.

After staining, slides were imaged using the Vectra 3.0 automated imaging system (PerkinElmer). First, whole-slide scans were made at 10x magnification. DCIS areas with surrounding stroma were annotated by a certified pathologist (MMA). Lymphocytes and macrophages beyond the limits of the DCIS lesion, surrounding normal ducts or around artifacts were excluded. Subsequently, multispectral image cubes of the entire DCIS area were acquired with 20x objective lens (0.5 micron/pixel) and processed to single images using HALO^®^ imaging analysis software (High-Plex FL v2.0 module, Indica labs). Multispectral library slides were created by staining a representative sample with each of the specific dyes. The multispectral library slides were unmixed into eight channels using InForm software version 2.4: DAPI, OPAL520, OPAL540, OPAL570, OPAL620, OPAL650, OPAL690 and Auto Fluorescence and exported to a multilayered TIFF file. The multilayered TIFF’s were fused with HALO software version 3.0 to create one file for each sample.

Image analysis was performed by a certified pathologist (M.M.A.) using HALO software (module HighPlex FL version 2.3.2089.69). Cells were phenotyped as CD3^+^ T-cells (all T-cells), CD20^+^ B-cells, CD3^+^CD8^−^ T-cells (helper T-cells, no co-expression of FOXP3), CD3^+^CD8^+^ T-cells (cytotoxic T-cells), CD3^+^FoxP3^+^ T-cells (regulatory T-cells), CD68^+^ cells (macrophages) or pancytokeratin AE1/AE3 positive (DCIS cells). Lymphocytes were defined as either CD3 or CD20 positive (lymphocytes = CD3^+^CD8^−^FOXP3^−^ T-cells + CD3^+^CD8^+^FOXP3^−^ T-cells + CD3^+^CD8^−^FOXP3^+^ T-cells + CD20^+^ B-cells). As CD4 (clone SP35) showed aspecific staining it was not included in the multiplex IF setting. Therefore, helper T-cells were defined as CD3^+^CD8^−^FOXP3^−^ T-cells. Cell phenotyping and tissue segmentation to differentiate between DCIS and stromal tissue compartments is further described in the supplementary methods. Immune cell density for each immune cell subset was assessed for the stromal and intraepithelial DCIS compartment and expressed as number of immune cells/mm^2^.

In order to study spatial associations between immune cells and DCIS ducts in the mIF setting, we developed the “DCIS proximity index” as novel metric for this study. The DCIS proximity index corresponds to the number of immune cells within 25 µm (approximate diameter) of a DCIS cell, divided by the total number of DCIS cells on the mIF images for each patient.

$${{{{{\mathrm{DCIS}}}}}}\;{{{{{\mathrm{proximity}}}}}}\;{{{{{\mathrm{index}}}}}} = \frac{{{{{{{{{\mathrm{number}}}}}}}}\;{{{{{{{\mathrm{of}}}}}}}}\;{{{{{{{\mathrm{immune}}}}}}}}\;{{{{{{{\mathrm{cells}}}}}}}}\;{{{{{{{\mathrm{within}}}}}}}}\;25\;{{\upmu {{{{{\mathrm{m}}}}}}}}\;{{{{{{{\mathrm{of}}}}}}}}\;{{{{{{{\mathrm{a}}}}}}}}\;{{{{{{{\mathrm{DCIS}}}}}}}}\;{{{{{{{\mathrm{cell}}}}}}}}}}{{{{{{{{{\mathrm{number}}}}}}}}\;{{{{{{{\mathrm{DCIS}}}}}}}}\;{{{{{{{\mathrm{cells}}}}}}}}}}$$

### Double-staining IHC of activated CD8^+^ T-cells

For the assessment of activated CD8^+^ T-cells, we evaluated the co-expression of CD8^+^ T-cells with Ki67. CD8^+^Ki67^+^ purple-yellow double IHC was performed on whole slides of the same set of 141 DCIS patients who were treated with BCS alone. Detailed information about the staining method, reagents and antibodies is provided in the supplementary methods.

Digital image analysis was performed using the HALO image analysis software, version 3.0.311.346 (Indica Labs). CD8^+^/Ki-67^+^ T-cells were quantified using the HALO multiplex algorithm version 2.0.3. Tissue segmentation was performed using HALO AI (CNN, VGG network). The tissue compartments “non-tumour”, “DCIS” and “intraluminal debris” used for training the tissue classifier were annotated as ground truth by S.V. and a certified pathologist (M.M.A.). Additionally, the percentage of Ki-67-positive DCIS cells was scored by two certified pathologists (M.M.A. and J.S.) that were blinded for outcome. To relate Ki-67 to the investigated IME factors, we used a predefined cutoff of 14% as described previously [10, 21, 22].

### Assessment of tertiary lymphoid structures (TLSs)

TLSs are ectopic lymphoid formations that are located in non-lymphoid tissues, including tumour sites. As TLSs have been associated with clinical benefit in cancer patients [23], we investigated if the presence of TLSs has prognostic value in outcome after primary DCIS. Different types or stages of TLSs have been reported. Sautès-Fridman et al. described that mature TLSs correspond to lymphoid follicles that include a dense cellular aggregate resembling a germinal centre. Lymphoid aggregates and lymphoid follicles without germinal centres were also defined as TLSs but they were considered less differentiated [23]. To our knowledge, it is not known if the presence or absence of germinal centres in TLSs is related to outcome in DCIS. Therefore we analysed TLSs in two ways 1) zone-TLSs: all TLSs consisting of CD20^+^ B-cell follicles adjacent to a CD3^+^ T-cell zone [23] as assessed on mIF images and 2) only TLSs with a germinal centre (GC-TLSs) as assessed on CD8/Ki67 double-staining IHC slides. GC-TLSs contain lymphoid follicles consisting of germinal centre B-cells positive for proliferation marker Ki-67.

Two pathologists assessed TLSs in the periductal stroma of DCIS lesions with CD20^+^ B-cell follicles adjacent to a CD3^+^ T-cell zone on mIF images (zone-TLS) and TLSs with Ki-67-positive germinal centres (GC-TLS) on CD8^+^Ki67^+^ double-staining IHC. Average TLS counts between the observers were compared between cases and controls. Pathologists were blinded for case or control status. Zone-TLS and GC-TLS density was defined as the number of TLSs/mm^2^.

### TILs assessment in DCIS

The percentage of stromal TILs was evaluated on H&E stained sections of FFPE tissues according to the criteria of the Immuno-Oncology Biomarker Working Group [10, 24]. Briefly, TIL percentage was considered both as a continuous and a categorical variable (in predefined groups of <1%, 1–49% and ≥50%). To assess the TIL score agreement between different H&E sections of the same patient, stromal percentage of TILs was scored by one breast pathologist on all available H&E slides of 20 women treated with BCS alone. Subsequently, stromal percentage of TILs was scored in one representative DCIS slide of all 141 DCIS patients treated with BCS alone by two pathologists. The average of the TILs scores of these two pathologists was calculated and correlated to stromal lymphocyte density as assessed by mIF. Average TILs scores were examined for their associations with subsequent iIBC.

### Statistics

We assessed immune cell density (cells per mm^2^) and immune cell composition within the periductal stromal tissue and within the DCIS epithelium. Immune cell composition was expressed as ratios between all available immune cell categories and subsets (all lymphocytes, CD3^+^ T-cells, CD3^+^CD8^−^ T-cells, CD20^+^ B-cells, CD3^+^FOXP3^+^ T-cells, CD3^+^CD8^+^ T-cells, CD8^+^Ki67^+^ activated T-cells, and CD68^+^ cells) and calculated for the periductal stromal and DCIS tissue compartments. All immune cell densities and ratios were compared between cases and controls.

Statistical significance of immune cell density and immune cell ratio associations was evaluated using the non-parametric Wilcoxon/Mann–Whitney *U*-test and Kruskal–Wallis test with permutation tests from the “coin” package in R. Statistical analyses were two-sided and nominal *P*-values < 0.05 were considered statistically significant. The Benjamini-Hochberg method was used for multiple testing correction in analyses resulting in nominally significant *P*-values, assessing significance at a false-discovery rate (FDR) less than 0.05.

Next, we examined if immune cell characteristics are associated with (1) grade, ER and HER2 status and (2) with markers that predict iIBC risk after primary DCIS (COX-2 and periductal fibrosis) [20]. We correlated stromal immune cell density with age, clinical presentation, time to iIBC, lesion size, margin status, dominant growth pattern, necrosis, calcifications, and PR. No multiple testing correction was performed for associations with clinicopathological DCIS characteristics, as these analyses validate expected patterns.

Unsupervised cluster analysis was carried out for both stromal and intraepithelial immune cell density using Euclidean distance on the log-transformed cell densities with complete linkage. Analyses were performed in R (version 4.0.3) using ggplot2, tidyverse coin and ComplexHeatmap packages.

Inter-observer agreement between two pathologists was assessed for zone-TLS, GC-TLS and TILs through the intraclass correlation coefficient (ICC). ICC estimates and their 95% confidence intervals were calculated using the irr and irrNA packages in R based on a mean-rating (*k* = 3), absolute-agreement, and two-way random-effects model. The ICC estimate and 95% CI was also calculated for DCIS TILs score agreement between different H&E slides within the same patient rated by one pathologist in *n* = 20 DCIS patients (two-way mixed effect model with single measurement). The Spearman correlation test was used to correlate average stromal TILs on H&E slides with stromal lymphocyte density as assessed with mIF.

## Results

### Multiplex IF analysis in DCIS patients

Clinical and histopathological characteristics of patients included in the study are shown in Table 1. Patients were derived from a Dutch population-based cohort of 10,090 patients diagnosed with pure DCIS between 1989 and 2005 with a median follow-up of 12.0 years (IQR 9.0–15.3), of which 2658 women were treated with BCS alone. Included patients were uniformly treated with BCS alone. Cases (*n* = 77) are women with DCIS that developed subsequent iIBC, while controls (*n* = 64) did not (Fig. 1). Median time to iIBC was 5.4 years among cases. Clinical and histopathological characteristics were comparable across cases and controls. As previously reported, COX-2 expression was significantly more frequent in cases compared to controls (*P* < 0.001).

**Table 1:** Clinical, histopathological and immunohistochemical characteristics of female primary DCIS patients treated with breast conserving surgery alone, who subsequently did (DCIS cases) or did not (DCIS controls) develop subsequent ipsilateral invasive breast cancer.

Characteristics	DCIS cases (*n* = 77)		DCIS controls (*n* = 64)		*P*
	*n*	(%)	*n*	(%)	
Clinical characteristics					
Age, mean (range)	57	(34–87)	58	(34–87)	0.61^a^
Age at DCIS diagnosis (years)					0.53^b^
≤50	17	(22)	11	(17)	
>50	60	(78)	53	(83)	
Year of DCIS diagnosis, median (range)	1996	(1989–2004)	1996	(1989–2004)	0.84^c^
Period of DCIS diagnosis					0.85^b^
1989-1998 (screening implementation phase)	57	(74)	49	(77)	
1999-2004 (full nation-wide coverage)	20	(26)	15	(23)	
Clinical presentation of DCIS					0.71^b^
Screen-detected	34	(45)	35	(55)	
Non-screening-related	5	(6)	3	(5)	
Unknown	38	(49)	26	(47)	
Time to iIBC, median in years (range)	5.4	(0.5–17.0)	-	-	
Histopathology					
Lesion size, millimetre mean (range)	13.9	(2–30)	10.9	(3–30)	0.14^c^
Lesion size					0.17^b^
≤10 mm	13	(17)	16	(25)	
>10 mm	16	(21)	8	(13)	
Unknown	48	(62)	40	(62)	
Margin status					0.35^b^
Free	38	(49)	31	(48)	
Not free	21	(27)	25	(39)	
Unknown	18	(23)	8	(13)	
Dominant growth pattern					0.44^d^
Clinging	5	(7)	1	(2)	
(Micro-)papillary	7	(9)	9	(14)	
Cribriform	17	(22)	13	(20)	
Solid	48	(62)	41	(64)	
Histologic grade					0.32^d^
Grade 1	12	(16)	5	(8)	
Grade 2	49	(64)	42	(66)	
Grade 3	16	(21)	17	(27)	
Necrosis					0.83^b^
Absent	15	(20)	14	(22)	
Present	62	(80)	50	(78)	
DCIS-associated calcifications					0.50^b^
Absent	15	(20)	9	(14)	
Present	62	(80)	55	(86)	
Periductal fibrosis					0.11^b^
Absent	55	(71)	44	(69)	
Present	22	(29)	20	(31)	
Immunohistochemistry					
ER^e^					0.83^b^
Negative	16	(21)	12	(19)	
Positive	60	(78)	52	(61)	
N/A	1	(1)	0	(0)	
PR^e^					0.49^b^
Negative	31	(40)	22	(34)	
Positive	45	(58)	42	(66)	
N/A	1	(1)	0	(0)	
HER2					1.00^b^
Negative	55	(71)	45	(70)	
Positive	21	(27)	18	(28)	
N/A	1	(1)	1	(2)	
Subtype					0.67^b^
HR+HER2−	53	(69)	42	(66)	
HR+HER2+	7	(9)	9	(14)	
HR-HER2+	14	(18)	9	(14)	
HR-HER2−	2	(3)	3	(5)	
N/A	1	(1)	1	(2)	
COX-2					0.001^b^
Low	1	(1)	11	(17)	
High	75	(97)	53	(83)	
N/A	1	(1)	0	(0)	
Ki-67^f^					0.59^b^
<14%	57	(74)	47	(73)	
≥14%	7	(9)	8	(13)	
N/A	13	(17)	9	(14)	

Clinical and histopathological characteristics and ER, PR, HER2 and COX2 status were already available [20].

*N/A* not assessable, *N/As* were not included in the analysis, *HR* hormone receptor, *HR +*  ER positive and/or PR positive, *HR*– ER negative and PR negative.

*P*-values of continuous variables were calculated as follows:

^a^Unpaired *T*-test.

^b^Fisher’s exact test.

^c^Mann–Whitney *U*-test.

^d^chi-square test.

^e^ER and PR were considered positive when ≥10% of luminal epithelial cells showed nuclear staining of any intensity.

^f^Ki67 was scored by two pathologists using the CD8^+^Ki67^+^ double IHC stainings. The concordance between the two pathologists was 79%, with an ICC of 0.72.

**Fig. 2:**
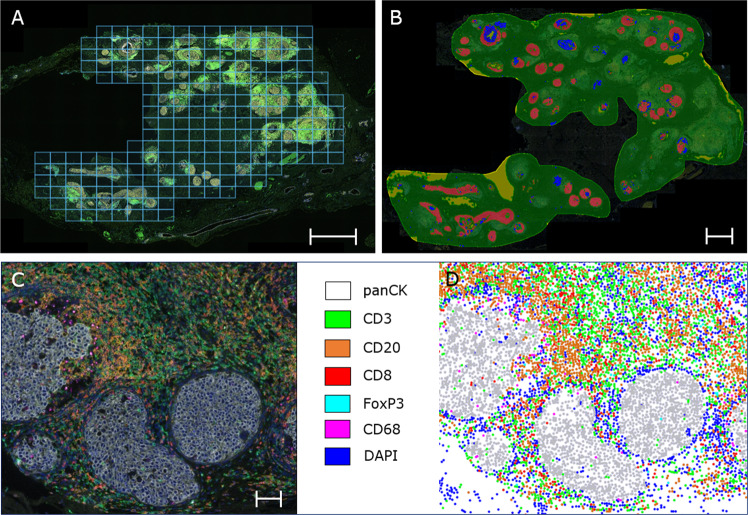
Pipeline mIF imaging of whole slides with DCIS. **A** Example of multispectral immunofluorescent overview scan (Phenochart) of whole slides with selection of DCIS areas for scanning at x200 magnification. 228 regions of interest (0.3345 mm^2^/tile) were scanned. The scalebar represents 1 mm. **B** We annotated the DCIS area and performed tissue segmentation (HALO^®^ software) dividing the scanned area in DCIS epithelium (red), periductal stroma (green), intraluminal debris (blue) and background (yellow) with a random forest classifier. The scalebar represents 1 mm. **C** High magnification of a DCIS lesion in which immune cells are predominantly present in the periductal tissue. Nuclei have been stained with DAPI, and DCIS ducts are pancytokeratin positive. The scalebar represents 100 µm. **D** Digital representation of DCIS cells (grey dots) and immune cells (spatial plot, HALO software, IndicaLabs). The blue dots represent nuclei of cells that are negative for pancytokeratin, CD3, CD20, CD8, FoxP3 and CD68.

Comprehensive automated tissue segmentation of DCIS lesions resulted in a total analysed area of 20,170 image fields (669 x 500 µm/image field) in all 141 patients, corresponding to a total area of 6747 mm^2^, with a median area of 38 mm^2^ (interquartile range (IQR) 16–70 mm^2^) per patient. After tissue segmentation, presence of immune cells was analysed in the intraepithelial DCIS and periductal stromal tissue compartments (Fig. 2).

### Stromal immune cell density is associated with DCIS characteristics

Multiplex IF analysis was successfully performed on whole slides of 141 DCIS patients. CD3, CD8, CD20, CD68, keratin AE1/AE3 and DAPI were assessable in all patients, while FoxP3 was assessable in 127 patients. Stromal lymphocyte density ranged from 10 to 2803 cells per mm^2^ (median 370 cells/mm^2^, interquartile range (IQR) 158–673 cells/mm^2^) among DCIS patients.

**Fig. 3:**
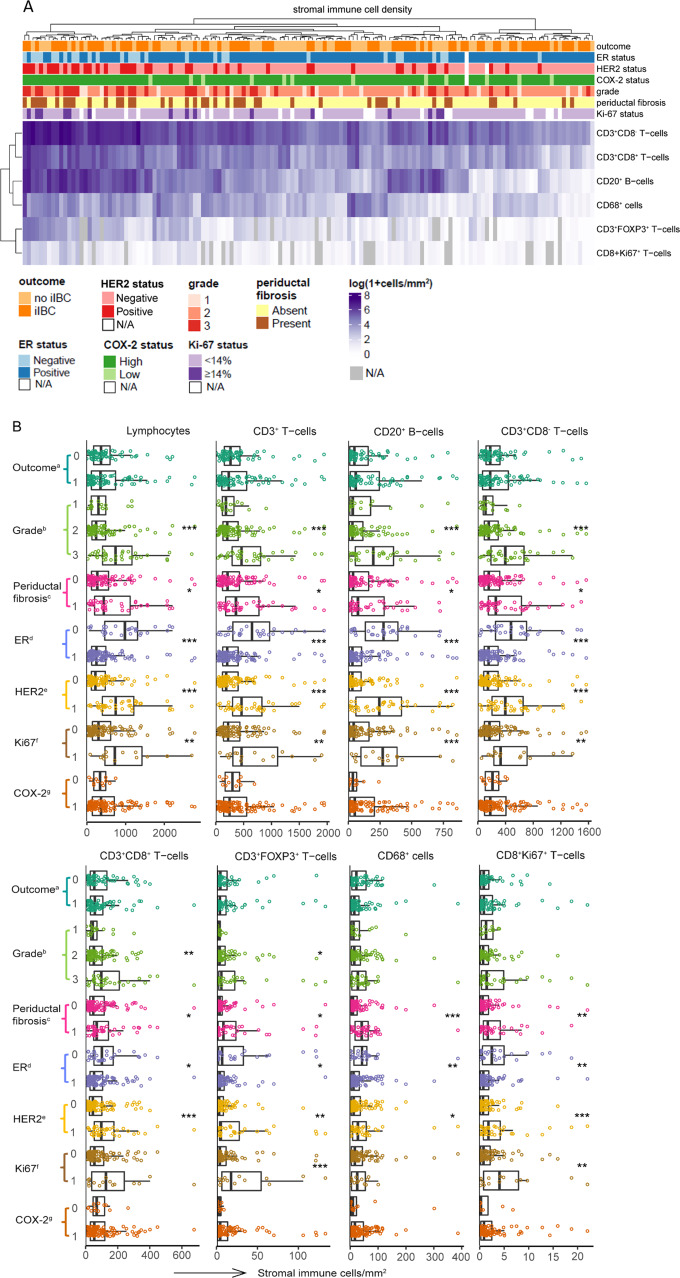
Immune cell density in the periductal stroma is associated with clinicopathological characteristics of DCIS. **A** Heatmap of log(1 + cells/mm^2^) stromal immune cell density of immune cell subsets related to outcome, grade, ER status, HER2 status, COX-2 expression and Ki67 in 141 DCIS patients. Stromal immune cell density does not cluster with case–control status. Higher stromal immune cell density clusters with higher grade, negative ER status, positive HER2 status, presence of periductal fibrosis and Ki67 ≥ 14%. Unsupervised cluster analysis was carried using Euclidean distance on the log-transformed cell densities with complete linkage. White rectangles: ER, HER2, COX-2, Ki67 not assessable (N/A). Grey rectangles: FoxP3^+^ T-cells and CD8^+^Ki67^+^ T-cells not assessable (N/A). **B** Combined beeswarm- and boxplots of stromal immune cell density. Higher stromal immune cell density is significantly associated with ER-negative status, HER2-positive status, presence of periductal fibrosis and Ki67 ≥ 14%. Higher grade is significantly associated with higher stromal density of lymphocytes, CD3^+^ T-cells, CD8^+^CD3^+^ T-cells, CD20^+^ B-cells and FoxP3^+^CD3^+^ regulatory T-cells (Kruskal–Wallis test). Significant associations are indicated as: *P < 0.05, **P < 0.01 and ***P < 0.001. The central line in boxes represent the median value, boundaries of boxes represent the interquartile range (IQR), and ends of whiskers represent values at 1.5x IQR. ^a^outcome, 0 = controls and 1 = cases, ^b^histologic grade was based on nuclear grade. ^c^periductal fibrosis, 0 = periductal absent, 1 = present. ^d^ER, 0 = negative, 1 = positive; ER was considered positive when ≥10% of the luminal epithelial cells showed nuclear staining of any intensity. ^e^HER2, 0 = negative, 1 = positive. ^f^Ki67 expression in DCIS cells, 0 = <14%, 1 = ≥14%. ^g^COX-2, 0 = low expression in DCIS cells, 1 = high expression in DCIS cells.

Higher stromal density of stromal lymphocytes, CD3^+^ T-cells, CD20^+^ B-cells, CD3^+^CD8^−^ T-cells, CD3^+^FOXP3^+^ T-cells, CD3^+^CD8^+^ T-cells, CD8^+^Ki67^+^ T-cells, and CD68^+^ cells was significantly associated with periductal fibrosis, negative ER status, negative PR status and positive HER2 status (Fig. 3B and Supplementary Table 3). Higher grade was significantly associated with higher stromal density of lymphocytes (*P* < 0.001), CD3^+^ T-cells (*P* < 0.001), CD20^+^ B-cells (*P* < 0.001), CD3^+^CD8^−^ T-cells (*P* < 0.001), CD3^+^CD8^+^ T-cells (*P* = 0.004) and CD3^+^FOXP3^+^ T-cells (*P* = 0.043). Ki67 ≥ 14% in DCIS cells was associated with higher stromal density of all immune cell subsets except for CD3^+^CD8^+^ T-cell and CD68^+^ cell density. Stromal immune cell density was not associated with COX-2 expression (Fig. 3B).

Upon analysing the relation between stromal immune cell density and all clinicopathological features we found that presence of comedonecrosis is significantly associated with higher lymphocyte, total T-cell, CD20^+^ B-cell, CD3^+^CD8^−^ T-cell and CD3^+^FOXP3^+^ T-cell density (*P* < 0.05, see Supplementary Table 3).

Density of analysed stromal immune cell subsets also significantly correlated with DCIS subtype. ER-negative/HER2-positive DCIS showed the highest stromal density for all immune cell subsets except for CD3^+^CD8^+^ T-cell density, while ER-positive/HER2-negative DCIS showed lowest stromal immune cell densities (Supplementary Table 4).

### Stromal density of analysed immune cell subsets is not associated with outcome

Importantly, stromal lymphocyte, CD3^+^ T-cell, CD20^+^ B-cell, CD3^+^CD8^−^ T-cell, CD3^+^FOXP3^+^ T-cell, CD3^+^CD8^+^ T-cell, CD8^+^Ki67^+^ T-cell, and CD68^+^ cell density did not differ between cases and controls (*P* > 0.05, Fig. 3). Taken together, we showed that stromal density of analysed immune cell subsets was associated with (immuno-)histopathological characteristics of primary DCIS, but not with the development of a subsequent iIBC.

### DCIS intraepithelial immune cell density is not associated with outcome

We also compared immune cell density in intraepithelial DCIS tissue. DCIS intraepithelial lymphocyte density varied from 0.00 to 234 lymphocytes/mm^2^ among all 141 DCIS patients (median 10.1 lymphocytes/mm^2^, IQR 5.0–21.1). Median DCIS intraepithelial density of lymphocytes, CD3^+^ T-cells, CD3^+^CD8^−^ T-cells, CD3^+^CD8^+^ T-cells, CD3^+^FOXP3^+^ T-cells and CD20^+^ B-cells was significantly lower than stromal immune cell density (*P* < 0.001, Supplementary Fig. 1 and Supplementary Table 5). DCIS intraepithelial CD68^+^ cell density (median 20.3 cells/mm^2^, IQR 8.0–50.6) and CD8^+^Ki67^+^ T-cell density (median 0.85 cell/mm^2^, IQR 0.21–2.87) did not differ from stromal CD68^+^ cell and CD8^+^Ki67^+^ T-cell density (*P* = 0.48 and *P* = 0.71, respectively). DCIS CD20^+^ B-cell intraepithelial immune cell density (median 0, IQR 0–0.2 cell/mm^2^) was strikingly low compared to stromal CD20^+^ B-cell density (median 50.7 cells/mm^2^, IQR 12.2–190). In the stromal tissue compartment, CD20^+^ B-cells comprised 13.7% of all lymphocytes, while in the intraepithelial DCIS compartment only 2.6% of lymphocytes were CD20^+^ B-cells (Supplementary Tables 3 and 5).

DCIS intraepithelial lymphocyte, CD3^+^ T-cell, CD3^+^CD8^−^ T-cell, CD3^+^FOXP3^+^ T-cell and CD8^+^Ki67^+^ T-cell density was significantly higher in HER2-positive DCIS compared to HER2-negative DCIS (Supplementary Fig. 1B). Higher intraepithelial CD3^+^FOXP3^+^ T-cell and CD8^+^Ki67^+^ T-cell density was significantly associated with ER-negative DCIS. Additionally, Ki67 ≥ 14% in DCIS cells was associated with higher CD3^+^FOXP3^+^ regulatory T-cell density.

DCIS intraepithelial lymphocyte, CD3^+^ T-cell, CD20^+^ B-cell, CD3^+^CD8^−^ T-cell, CD3^+^FOXP3^+^ T-cell, CD3^+^CD8^+^ T-cell, CD8^+^Ki67^+^ T-cell, and CD68^+^ cell density did not differ between cases and controls (Supplementary Fig. 1). In general, DCIS intraepithelial immune cell density is lower than stromal immune cell density and not related to outcome. Correlations of intraepithelial immune cell density with (immuno-)histopathological features are less outspoken compared to stromal immune cell density.

### Relation of stromal and intraepithelial immune cell ratios and outcome

Calculation of ratios between all analysed immune cell subsets resulted in 28 different combinations. None of the immune cell ratios for stromal and intraepithelial immune cell subsets differed between cases and controls (Supplementary Table 6, false-discovery rate (FDR) => 0.05). Therefore, the composition of analysed immune cells was not related to subsequent iIBC risk.

### Case–control comparisons in subgroups

As grade, ER, HER2, periductal fibrosis and comedonecrosis were significantly associated with stromal immune cell density, we assessed the relation with outcome in the largest subgroups (the ER^+^/HER2^–^ subgroup and the subgroup in which periductal fibrosis was absent). The other subgroups contained insufficient patient numbers.

Within the ER^+^/HER2^−^ subgroup (*n* = 95), stromal CD3^+^FOXP3^+^ T-cell density was higher in controls (*n* = 42) (median 4.25 cells/mm^2^, IQR 2.04–9.17 cells/mm^2^) compared to cases (*n* = 53) (median 1.42 cells/mm^2^, IQR 0.30–5.99 cells/mm^2^, nominal *P* = 0.034), but this was not significant after multiple testing correction (FDR > 0.05, Supplementary Table 7). Stromal lymphocyte, CD3^+^ T-cell, CD20^+^ B-cell, CD3^+^CD8^–^ T-cell, CD3^+^CD8^+^ T-cell, CD8^+^Ki67^+^ T-cell, and CD68^+^ cell density did not differ between cases and controls (*P* > 0.05). Intraepithelial DCIS density was similar among cases and controls for all immune cell subsets (*P* > 0.05, Supplementary Table 7).

The presence of periductal fibrosis could interfere with the infiltration of immune cells. Therefore, we analysed stromal and DCIS intraepithelial lymphocyte, CD3^+^ T-cell, CD20^+^ B-cell, CD3^+^CD8^−^ T-cell, CD3^+^FOXP3^+^ T-cell, CD3^+^CD8^+^ T-cell, CD8^+^Ki67^+^ T-cell, and CD68^+^ cell density in relation to outcome among all women with DCIS in whom periductal fibrosis was absent (*n* = 98). In the periductal fibrosis-absent subgroup, neither stromal nor DCIS intraepithelial immune cells of all subtypes differed between 55 cases and 43 controls (*P* > 0.05, Supplementary Table 7).

### Multiplex IF staining for myeloid cells

To further expand on using the CD68 marker for exploring the myeloid cell compartment, we profiled a small subset of controls (*n* = 11) and cases (*n* = 15) from MD Anderson Cancer Centre (*n* = 8) and the Dutch nation-wide study (*n* = 18) for tumour-associated macrophages (TAM2), granulocytic myeloid-derived suppressor cells (G-MDSC’s), myeloid-derived suppressor cells (M-MDSC’s), macrophages, granulocytes and monocytes (Supplementary Methods, Supplementary Fig. 3a–c and Supplementary Table 8). The myeloid multiplex IF panel revealed no significant difference in the myeloid cell frequencies between cases and controls (Supplementary Fig. 3d).

### Tertiary lymphoid structures (TLS) in DCIS

**Fig. 4:**
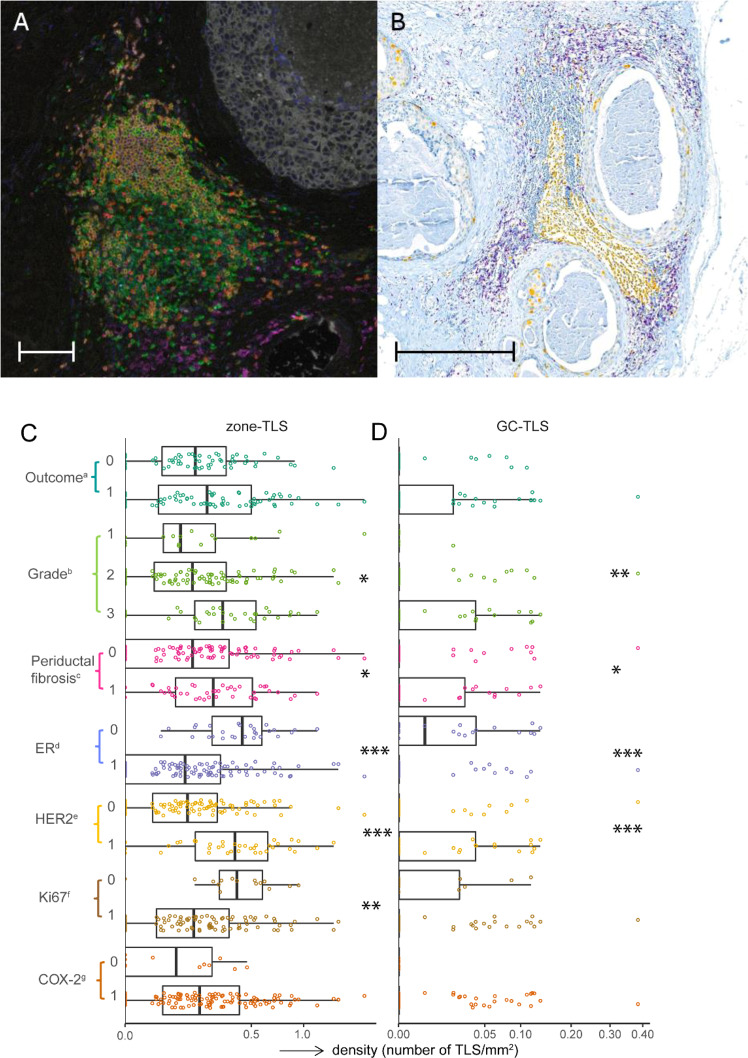
TLSs with and without a germinal centre in DCIS. **A** Multispectral IF image of a DCIS duct with adjacent zone-TLS consisting of a CD20^+^ B-cell follicle surrounded by a CD3^+^ T-cell zone. Orange: CD20, green: CD3, red: CD8, white: keratinAE1/AE3, magenta: CD68, turquoise: FOXP3, and blue: DAPI. The scalebar represents 100 µm. **B** CD8^+^Ki67^+^ double-staining IHC of DCIS lesion with large GC-TLS containing a Ki-67-positive germinal centre. Yellow: Ki-67, purple: CD8. The scalebar represents 500 µm. **C**, **D** Combined beeswarm- and boxplots with zone-TLS (**C**) and GC-TLS density (**D**) in relation to outcome, ER status, HER2 status, periductal fibrosis, Ki67 and COX2 expression (Wilcoxon–Mann–Whitney test) and grade (Kruskal–Wallis test). Significant associations are indicated as: *P < 0.05, **P < 0.01 and ***P < 0.001. The central line in boxes represent the median value, boundaries of boxes represent the interquartile range (IQR), and ends of whiskers represent values at 1.5x IQR. ^a^outcome, 0 = controls and 1 = cases. ^b^histologic grade was based on nuclear grade. ^c^periductal fibrosis, 0 = periductal fibrosis absent, 1 = present. ^d^ER, 0 = negative, 1 = positive; ER was considered positive when ≥10% of the luminal epithelial cells showed nuclear staining of any intensity. ^e^HER2, 0 = negative, 1 = positive. ^f^Ki67 expression in DCIS cells, 0 = <14%, 1 = ≥14%. ^g^COX-2, 0 = low expression in DCIS cells, 1 = high expression in DCIS cells.

We investigated if the presence of zone-TLS and/or GC-TLS has prognostic value in outcome after primary DCIS in the Dutch nation-wide DCIS series. Zone-TLS were assessed on multiplex IF images (*n* = 141) and consisted of a CD3^+^ T-cell zone and a CD20^+^ B-cell zone. GC-TLS were characterised by lymphocytic aggregates with a Ki67-positive germinal centre and visualised on CD8/Ki67 double-staining IHC images (*n* = 118). Figure 4 shows examples of a zone-TLS and a GC-TLS.

The concordance of zone-TLS assessment between two pathologists was 84% with an intraclass correlation coefficient (ICC) of 0.89 (95% CI 0.85–0.92, interpreted as good to excellent agreement). The concordance of GC-TLS was 95% with an ICC of 0.97 (95% CI 0.96–0.98, interpreted as excellent agreement), respectively.

Median zone-TLS density (number of zone-TLS/mm^2^) was 0.17 TLS/mm^2^ (IQR 0.04–0.39) while median GC-TLS density was 0 GC-TLS/mm^2^ (IQR 0.00–0.47 TLS/mm^2^). Density of zone-TLS and GC-TLS did not differ between cases and controls. Higher zone-TLS density was associated with positive HER2 status (*P* < 0.001), negative ER status (*P* < 0.001), presence of periductal fibrosis (*P* = 0.040), higher grade (*P* = 0.032) and Ki67 ≥ 14% (*P* = 0.002). Similar associations were found for higher GC-TLS density (Fig. 4).

### Spatial relationships and outcome

The number of immune cells within a distance of 25 µm of a DCIS cell divided by total number of DCIS cells was expressed as DCIS proximity index and assessed for all immune cell subsets on mIF images. Median DCIS proximity index for lymphocytes was 0.013 cells per DCIS cell (IQR 0.005–0.033). DCIS proximity index for lymphocytes, CD3^+^ T-cells, CD20^+^ B-cells, CD3^+^CD8^−^ T-cells, CD3^+^FOXP3^+^ T-cells, CD3^+^CD8^+^ T-cells and CD68^+^ cells per DCIS cell did not differ between cases and controls (Supplementary Fig. 2, *P* > 0.05).

### DCIS TILs assessment on H&E slides

DCIS TILs scores between different H&E slides of the same DCIS patient in a subset of 20 patients were similar (see Supplementary Table 9). Among 141 DCIS patients, 5 women (3.5%) had <1%, 131 women (93%) 1–49%, and 5 women (3.5%) ≥50% periductal stromal lymphocytes on H&E slides (see Supplementary Table 10). At the continuous scale, stromal TILs on H&E slides correlated with stromal lymphocytes/mm^2^ as assessed with mIF (*ρ* = 0.71, *P* < 0.001). Median stromal TILs was 7.5% in cases and controls. Stromal TILs on H&E slides were not associated with increased risk for subsequent iIBC (Supplementary Table 10). Low or no TILs (defined as stromal TILs below a threshold of 1% or 5%) neither protected against subsequent iIBC nor increased the risk.

## Discussion

In the present study, we show that analysed factors from the DCIS immune microenvironment (IME) are no independent predictors for risk of subsequent iIBC after diagnosis of primary, pure DCIS. The investigated IME factors, however, do correlate with factors (ER, HER2, Ki-67, grade and periductal fibrosis) that are known to be associated with invasive recurrences. Our results have unique new and added value for the following three main reasons. First, the results are based on a large and well-annotated DCIS patients series derived from a Dutch, nation-wide cohort with long-term follow-up. Second, the IME was analysed by in-depth multiplex immunofluorescence. Third, whole slides were analysed, preventing that IME heterogeneity compromises the reliability of the results, as is more likely to occur if our analysis had to rely on a limited number of regions of interest. The validity of our approach is clear from the fact that in our study high stromal density of lymphocytes, CD3^+^CD8^−^ T-cells, CD3^+^CD8^+^ T-cells, CD3^+^FOXP3^+^ T-cells, CD20^+^ B-cells and CD68^+^ macrophages is associated with ER negativity, HER2 positivity and periductal fibrosis. Higher stromal immune cell density was also associated with higher grade for all analysed immune cell subsets except for CD68^+^ macrophages. Our finding that higher prevalence of stromal immune cell subsets is associated with higher DCIS grade, ER negativity and HER2 positivity is in concordance with previous studies [10, 15, 18, 25–28]. Grade, ER and HER2 status are, however, easily assessed with light microscopy and immunohistochemistry.

We did not find evidence that the analysed immune parameters predict subsequent iIBC after pure primary DCIS treated with BCS alone. In particular, we find that density, composition and distance to the closest DCIS cell of lymphocytes, CD3^+^ T-cells, CD20^+^ B-cells, CD3^+^CD8^−^ T-cells, CD3^+^CD8^+^ T-cells, CD3^+^FOXP3^+^ T-cells, CD8^+^Ki67^+^ activated T-cells and CD68^+^ macrophages do not differ between DCIS patients that developed subsequent iIBC versus DCIS patients that did not.

The absence of a correlation between outcome and total periductal lymphocytes as assessed with both mIF and on H&E slides by pathologists is in line with the study of Pruneri et al. Percentage of TILs in the periductal stroma was scored by pathologists on H&E slides according to the criteria of the International Immuno-Oncology Biomarker Working Group. Among 1488 DCIS patients, TILs percentage did not differ between 136 patients that developed iIBC versus DCIS patients that did not have a recurrence [10]. As the patients were not stratified according to the treatment of their primary DCIS (BCS, mastectomy, RT and/or endocrine therapy), it cannot be excluded from this study that treatment impacted the outcome.

Gil Del Alcazar et al. suggested that the frequency of activated CD8^+^ T-cells may predict which DCIS lesions are likely to progress to IBC [19]. The study showed enrichment in activated effector CD8^+^ T-cells characterised by the expression of granzyme B (GZMB) or Ki67 in DCIS. Specifically, higher frequency of CD8^+^Ki67^+^ and CD8^+^GZMB^+^ T-cells was detected in the primary DCIS lesions of four patients compared to the subsequent IBC counterparts. In our study, we find no difference of CD8^+^Ki67^+^ T-cell density between 64 cases and 53 controls. Our data do not support the hypothesis that the frequency of activated CD8^+^Ki67^+^ T-cells predict subsequent iIBC.

We find no difference in “DCIS proximity index” (number of immune cells per DCIS cell within a distance of 25 µm divided by total number of DCIS cells) between cases and controls for all immune cell subsets. Toss et al. found a statistically significant association between high number of TILs (≥20) touching the DCIS basement membrane and a shorter recurrence-free survival in pure DCIS [18]. This association was also found in a subset of patients treated with BCS with or without RT (*n* = 219 with 84 local recurrences). The authors did not distinguish between a subsequent iIBC or subsequent ipsilateral DCIS according to their definition of a local recurrence, whereas in our study we uniformly included patients with subsequent iIBC. Differences in patient characteristics and assessment methodology could explain the discrepancy between both studies. The DCIS proximity index studies immune cells that are in close proximity to DCIS epithelial cells, while the touching TILs are lymphocytes that touch/are in close proximity to the basement membrane. In order to reproduce the touching TILs method more closely in the context mIF, a future study may include a mIF panel consisting of immune cell markers and collagen IV to highlight the basement membrane, allowing a more accurate evaluation of immune cells in relation to the basement membrane. Future research may further explore co-localisation of immune cells [29] with the DCIS epithelial cells.

While most studies focus on TILs, Chen et al. found a worse DFS for subsequent iIBC (*n* = 27) among 198 DCIS patients with a higher CD163^+^ and CD68^+^ macrophage density [17]. In our study, neither stromal nor intraepithelial CD68^+^ cell density was associated with subsequent iIBC. A principal difference between both studies is that DCIS patients in the study of Chen et al. were treated with mastectomy or BCS with RT. Our exploratory analysis of the myeloid cell compartment in a subset of 26 DCIS patients also did not reveal differences between cases and controls.

Previously, Toss et al. showed that lymphocytic aggregates and lymphoid follicles do not have prognostic value [18]. In our study, density of zone-TLS consisting of CD20^+^ B-cell follicles adjacent to a CD3^+^ T-cell zone in mIF and GC-TLS containing a germinal centre as assessed on CD8/Ki67 double-staining IHC slides also did not predict subsequent iIBC. To further aid in the detection of GCs in TLSs, future studies may include CD21, CD23, and CD35 to highlight follicular dendritic cells and follicular dendritic networks, and activation-induced deaminase (AID), BCL-6 and CD269 for germinal centre B-cells [23].

A wide range of other immune parameters in relation to recurrence have been previously reported [11–14, 30]. Many studies are limited by small patient series, lack of treatment stratification or do not differentiate between invasive and in situ recurrences. As breast cancer mortality is only increased in DCIS patients that develop IBC [31], it is important to distinguish invasive from in situ recurrences, especially in the context of preventing overtreatment. Furthermore, as treatment confounds the association between prognostic markers and outcome, treatment needs to be accounted for.

Several other important findings emerged from the current study. Although higher TIL density previously has been associated with younger age [12, 18], symptomatic presentation [18] and larger size [12, 18], this was not confirmed in our study. The present study showed that comedonecrosis is associated with higher density of stromal lymphocytes, CD3^+^ T-cells, CD3^+^CD8^−^ T-cells, CD3^+^CD8^+^ T-cells, and CD20^+^ B-cells. Previous studies reported that a higher TIL and CD3^+^CD8^+^ T-cell density was associated with comedonecrosis [12, 18, 32].

A major strength of the study is the use of a large, unbiased and well-annotated sample series of women with pure DCIS treated with BCS alone that did or did not develop iIBC during long-term follow-up. None of the DCIS patients received RT and/or endocrine therapy that could impact biology of the ipsilateral residual breast tissue. This provided a unique opportunity for in-depth mIF and double IHC analysis in a well-designed case–control study to assess whether immune parameters relate to risk of transition to iIBC after DCIS.

Second, the entire spectrum of DCIS lesions was included in the study. There was no selection for higher DCIS grade lesions or DCIS lesions with a moderate or severe immune infiltrate. Multiplex IF analysis allowed for the study of different immune cell subsets while using whole slides, hence both large and small DCIS lesions were included.

Third, immune cell density was expressed as number of immune cells per millimetre rather than percentage of TILs. Immune cell density per millimetre may be a more objective measure since it is less likely confounded by the cellularity of fibroblasts, endothelial cells, adipocytes or other cells.

Finally, the use of specific, robust and validated antibodies with additional singleplex IF validation was crucial for achieving a successful mIF panel. Multiplex IF with digital image analysis allowed for marker co-localisation and synchronous-level quantification of immune cells, study of the composition of immune cells and spatial information. To our knowledge, this is the largest mIF study in which DCIS on whole slides was analysed resulting in a total amount of 20,170 image fields (669 x 500 µm/image field) in 141 patients.

Our study has some limitations. The findings in this study mainly apply to DCIS that is grade 2, solid type, associated with calcifications, ER+/HER2–, Ki67 < 14% and in patients that are >50 years old (with unknown clinical presentation in nearly 50%). Lesion size and margin status were unknown in 62% and 18% of patients, because these parameters were not commonly reported in the older retrospective series. Yet, among DCIS patients with known lesion size and margin status, these parameters did not differ between cases and controls. Moreover, missing data were equally distributed among these groups (Table 1). Therefore, a relation with outcome is highly unlikely in this patient series.

Secondly, multiplex IF Vectra^®^ 3.0 technology required extensive method validations. As described in literature, common variations in tissue structure and cell morphology resulted in lack of a generalised algorithm [33]. To overcome this limitation, semi-automated image analysis in HALO required manual curation by a certified pathologist to ensure accurate digital tissue/cell segmentation and cell phenotyping. To further safeguard accurate interpretation of imaging results, postprocessing corrections were performed to correct for the “bleed through” between the fluorescent channels, an intrinsic feature of the Vectra 3.0 platform.

Thirdly, we only investigated immune parameters for which reproducible and robust antibodies were available in the mIF setting. We may have overlooked other immune parameters with prognostic value for subsequent iIBC that we did not include in the study. Antibodies that did not pass our quality assessment for multiplex IF were CD4 (clone SP35), CD138 (clone B-A38), CD15 (clone MMA), Neutrophil elastase (clone SP203), Granzyme B (clones 11F1, GrB-7), CD56 (clone MRQ-42) and CD16 (clone SP175) due to background staining and non-specificity. Other promising avenues to gain deeper insight in the IME include multiplexed imaging such as CO-Detection by indexing (CODEX) [34], Digital Spatial Profiling technology [35] and MIBI-TOF (multiplexed ion beam imaging by time of flight) [36] allowing detection of a higher number of proteins. However, these techniques are often restricted to analyse a limited area, rather than whole slides.

The lack of a correlation between immune factors and outcome after DCIS in our study could be explained by the fact that among patients with iIBC after DCIS, in 18% the subsequent iIBC may be clonally unrelated to the primary DCIS [37]. It was estimated, however, that the majority (75%) of DCIS-subsequent iIBC pairs were clonally related. Further studies may unravel the relation of the IME with subsequent iIBC while taking clonal relatedness into account. Finally, transition to iIBC after DCIS may be caused by factors other than the IME, such as the DCIS epithelium (HER2 and COX-2 expression), and breast adipocyte size [20, 38].

Further studies are needed to investigate whether the findings of this study also apply to DCIS patients that receive radiotherapy after BCS. However, the role of the IME in DCIS progression should ideally be assessed in a prospective cohort with long-term follow up in which the biological course of DCIS is not influenced by treatment. The LORIS (United Kingdom, NCT02766881) [39], COMET (United States of America, NCT02926911) [40] and LORD (The Netherlands, NCT02492607) [41] trials compare active surveillance with conventional in DCIS patients with low-risk DCIS (grade 1 and/or grade 2 and additional inclusion criteria depending on the trial). Although high-risk DCIS is not included in these studies, it would be invaluable to investigate the relation of the IME with outcome in diagnostic biopsies from DCIS patients of the active surveillance arms.

In conclusion, we provide a comprehensive in-depth multiplex IF analysis in a large, well-annotated DCIS patient series with long-term follow-up. Multiplex IF did not reveal immune parameters with predictive value for iIBC risk after pure primary DCIS treated with BCS alone. It remains to be seen whether other approaches, such as single cell sequencing of DCIS and the IME may reveal biological and prognostic value in distinguishing harmless from hazardous DCIS [42]. Alternatively, integration of genomic and transcriptomic data of the DCIS epithelium with immune profiling data may shed more light on their interplay in the context of progression to iIBC and mechanisms of potential immune escape.

## Supplementary information

supplemental_material

REMARK checklist

## Data Availability

The data that support the findings of this study are available in a figshare repository (10.6084/m9.figshare.19698286).

## Code availability

The code for data that support the findings of this study is available at the GitHub repository https://github.com/NKI-CCB/precision-immune-analyses.
