# Supplementary material for: Comprehensive multiplexed immune profiling of the ductal carcinoma in situ immune microenvironment regarding subsequent ipsilateral invasive breast cancer risk
Source: Br J Cancer. 2022 Jun 29;127(7):1201–13. doi: 10.1038/s41416-022-01888-2 (PMC9519539; doi:10.1038/s41416-022-01888-2)
Supplement: Supplementary file 1 — supplemental_material [file 41416_2022_1888_MOESM1_ESM.docx]

**Supplementary information**

**Supplementary Materials and Methods**

Staining and analysis of automated multispectral imaging

For each patient from the Dutch case-control study, one 3 µm freshly cut representative formalin-fixed paraffin embedded whole tissue section with pure primary DCIS on DAKO Flex IHC slides was dried overnight and stored at 4 °C. Prior to multiplex staining, the slides were incubated at 70 °C. Staining was performed on a Ventana Discovery Ultra automated stainer, using the Opal 7-Color Manual IHC Kit (50 slides kit, Perkin Elmer, catalogous number (cat) NEL81101KT). Protocol starts with baking for 28 minutes at 75°C, followed by dewaxing with Discovery Wash using the standard setting of 3 cycles of 8 minutes at 69°C. Pretreatment was performed with Discovery CC1 buffer for 32 minutes at 95°C, after which Discovery Inhibitor was applied for 8 minutes to block endogenous peroxidase activity. Specific markers were detected consecutively on the same slide with the following antibodies (Supplementary Table 1), Anti-CD3 (SP7, Cat M3074, SpringBio, 1/400 dilution 1 hour at RT), anti-CD8 (Clone C8/144B, Cat M7103,DAKO, 1/100 dilution 1 hour at RT), anti-CD68 (Clone KP1, M0814, Dako, 1/500 dilution, 1 hour at RT), anti-FoxP3 (clone 236A/47, Cat ab20034, Abcam, 1/50 dilution, 2 hour at RT), Anti-CD20 (Clone L26, cat M0755, Dako, 1/500 dilution, 1 hour at RT), Anti-PanCK (Clone AE1AE3, Cat MS-343P, Thermo Scientific, 1/250 dilution, 2 hour at RT). Each staining cycle was composed of four steps: Primary Antibody incubation, Opal polymer HRP Ms+Rb secondary antibody incubated for 32 minutes at RT, OPAL dye incubation (OPAL520, OPAL540, OPAL570, OPAL620, OPAL650, OPAL690, 1/50 or 1/75 dilution as appropriate for 32 minutes at RT) and an antibody denaturation step using CC2 buffer for 20 minutes at 95°C. Cycles were repeated for each new antibody to be stained. At the end of the protocol slides were incubated with DAPI (1/25 dilution in Reaction Buffer) for 12 minutes. After the run was finished slides were washed with demi water and mounted with Fluoromount-G (SouthernBiotech, cat 0100-01) mounting medium.

The intensity of each fluorescent marker was normalized in comparison to batch controls. Thresholding for all markers with histopathological correlation was performed in order to accurately phenotype cells. Despite extensive optimization of the automated multiplex IF panel and meticulous thresholding in HALO, spectral overlap occurred. Each image was carefully analysed for spectral overlap by a certified pathologist (M.M.A.). Raw data needed post-processing corrections for spilling-through of immunofluorescent signal as summarized in supplementary table 2.

We trained supervised machine learning algorithms (random forest classifiers) for tissue segmentation to differentiate between DCIS and stromal tissue compartments. The morphological variation in tissue structure between different patients necessitated the use of multiple random forest classifiers.

Accurate tissue and cell segmentation was assessed by a certified pathologist and achieved for multiplex images of every patient, using 75 custom-made random forest classifier algorithms and 99 cell segmentation algorithms, respectively.

For CD8/Ki67 double-staining IHC, images of 22 patients were fully annotated and trained within the HALO AI (CNN, VGG network), resulting in accurate tissue segmentation of all 118 patients.

Double-staining IHC of CD8^+^/Ki-67^+^ T-cells

Double-staining IHC of the FFPE DCIS samples was performed on a Discovery Ultra autostainer. Briefly, paraffin sections were cut at 3 um, heated at 75°C for 28 minutes and deparaffinised in the instrument with EZ prep solution (Ventana Medical Systems). Heat-induced antigen retrieval was carried out using Cell Conditioning 1 (CC1, Ventana Medical Systems) for 64 minutes at 95^0^C. For the double staining Ki67 (Yellow) followed by CD8 (Purple) the Ki67 was detected in the first sequence using clone MIB1 (1/100 dilution, 1 hour at 37^0^C, Agilent / DAKO).

Ki67 bound antibody was visualized using Anti-Mouse NP (Ventana Medical systems) for 12 minutes at 37^0^C followed by Anti-NP AP (Ventana Medical systems) for 12 minutes at 37^0^C, followed by the Discovery Yellow detection kit (Ventana Medical Systems). In the second sequence of the double staining procedure CD8 was detected using clone C8/144B (1:200 dilution, 32 minutes at 37^0^C, Agilent / DAKO). CD8 was visualized using Anti-Mouse HQ (Ventana Medical systems) for 12 minutes at 37^0^C followed by Anti-HQ HRP (Ventana Medical systems) for 12 minutes at 37^0^C, followed by the Discovery Purple Detection Kit (Ventana Medical Systems). Slides were counterstained with Hematoxylin and Bluing Reagent (Ventana Medical Systems).

Multiplex immunofluorescence (IF) of myeloid cells

For the study of myeloid-derived suppressor cells (MDSC) multiplex IF was performed using similar methods that have been previously described and validated^1^ on a small subset of DCIS patients from the Dutch nation-wide study (n=18) and MD Anderson Cancer Center (n=8), (supplementary table 8). Briefly, 4 µm-thick FFPE sections were stained using an automated staining system (BOND-RX; Leica Microsystems, Buffalo Grove, IL) using a panel containing antibodies against CD68, CD11b, CD33, Arg-1, CD66b, CD14, panCK and DAPI to characterize tumour-associated macrophages (TAM2), granulocytic MDSC’s (G-MDSC), monocytic MDSC’s (M-MDSC) using colocalization of markers (supplementary table 1c and supplementary figure 3a-c).

All the markers were stained in sequence using their respective fluorophore containing in the Opal 7 kit (catalogue #NEL797001KT; Akoya Biosciences/PerkinElmer, Waltham, MA) and the TSA fluorophore Opal Polaris 480 (#FP1500001KT, Akoya Biosciences) was added to the kit. The stained slides were scanned using the multispectral microscope, Vectra 3.0 imaging system (Akoya Biosciences/PerkinElmer PerkinElmer), at low magnification (10x).^2^ After scanning at low magnification, a pathologist (FY and ERP) selected around five ROI (each ROI, 0.3345 mm^2^) per sample to cover different features in the DCIS epithelium and surrounding stromal tissue using the phenochart 1.0.9 viewer (Akoya Biosciences/PerkinElmer), supplementary figure 3a. The ROIs were analysed by a pathologist using InForm 2.8.2 image analysis software (Akoya Biosciences/PerkinElmer PerkinElmer).

Study approval

The Institutional Review Board (IRB) at the University of Texas MD Anderson Cancer Center provided approval for the use of the 8 MDA patient samples and associated clinical data, as part of protocol PA17-1020. A waiver of informed consent requirements was granted by the IRB as set forth in the U.S. Code of Federal Regulations (45 CFR 46.116).

**Supplementary consortium details**

Grand Challenge PRECISION Consortium Steering Group

Jelle Wesseling (Netherlands Cancer Institute, Amsterdam, The Netherlands), Alastair Thompson (Baylor College of Medicine, Houston, Texas, USA), Serena Nik-Zainal (University of Cambridge, Cambridge, UK), Elinor J. Sawyer (King’s College London, London, UK), Helen Davies (University of Cambridge, Cambridge, UK), Andrew Futreal (MD Anderson Cancer Center, Houston, USA), Nicholas Navin (MD Anderson Cancer Center, Houston, USA), E. Shelley Hwang (Duke University School of Medicine, Durham, NC, USA), Jos Jonkers (Netherlands Cancer Institute, Amsterdam, The Netherlands), Jacco van Rheenen (Netherlands Cancer Institute, Amsterdam, The Netherlands), Fariba Behbod (Kansas University Medical Center, Kansas, USA), Esther H. Lips (Netherlands Cancer Institute, Amsterdam, The Netherlands), Marjanka Schmidt (Netherlands Cancer Institute, Amsterdam, The Netherlands), Lodewyk F.A. Wessels (Netherlands Cancer Institute, Amsterdam, The Netherlands), Daniel Rea (University of Birmingham, Birmingham, UK), Proteeti Bhattacharjee (Netherlands Cancer Institute, Amsterdam, The Netherlands), Hilary Stobart (Independent Cancer Patients' Voice, UK), Deborah Collyar (Patient Advocates in Research, USA), Donna Pinto (dcis411, USA), Ellen Verschuur (Borstkanker Vereniging Nederland, The Netherlands), Marja van Oirsouw (Borstkanker Vereniging Nederland, The Netherlands).

**Supplementary figures and tables**


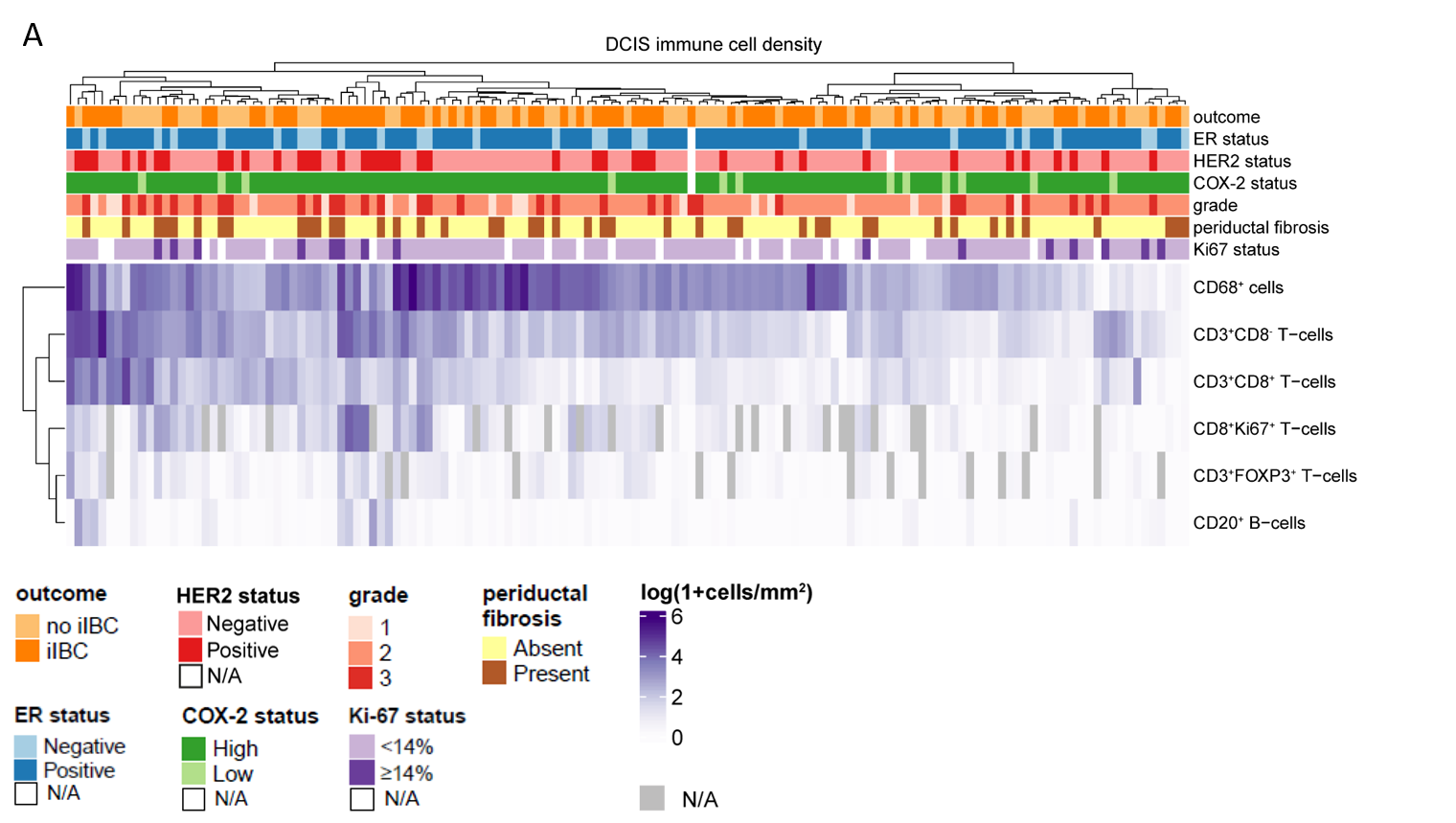


**
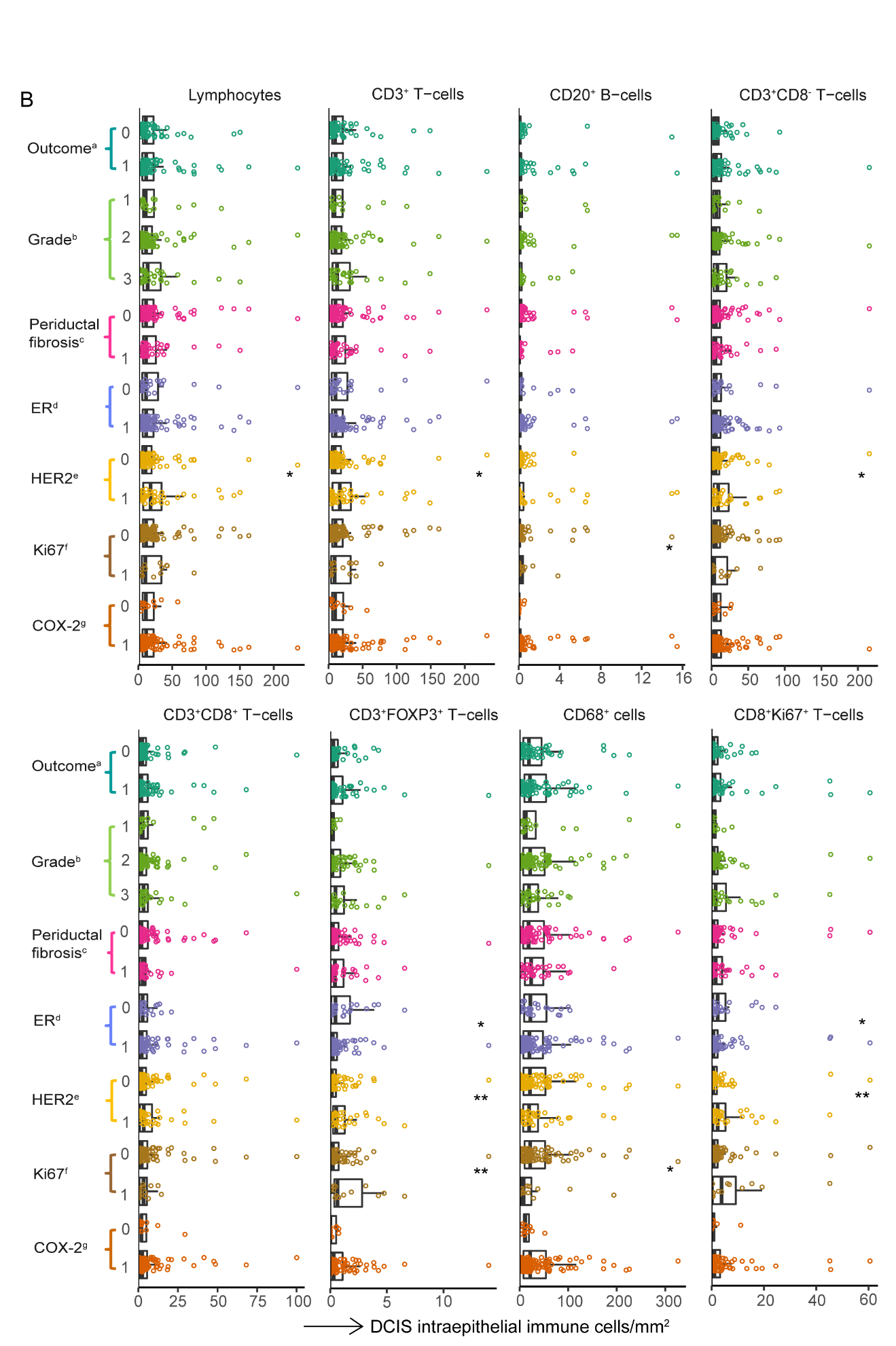
Supplementary figure 1. Immune cell density in the DCIS epithelium is not associated with risk of subsequent iIBC**

A. Heatmap of log(1+cells/mm^2^) immune cell density within the DCIS epithelium of CD3^+^CD8^-^ T-cells, CD8^+^ T-cells, CD20^+^ B-cells, CD68^+^ cells, CD3^+^FoxP3^+^ T-cells and CD8^+^Ki67^+^ T-cells related to outcome, grade, ER status, HER2 status, Ki67 and COX-2 expression in 141 DCIS patients. DCIS intraepithelial immune cell density is lower than stromal immune cell density and is not associated with outcome. Unsupervised cluster analysis was carried using Euclidean distance on the log-transformed cell densities with complete linkage.

B. Combined beeswarm- and boxplots of DCIS immune cell density. Higher DCIS intraepithelial CD3^+^FoxP3^+^ regulatory T-cell and CD8^+^Ki67^+^ T-cell density is significantly associated with negative ER status, whereas higher intraepithelial lymphocyte, CD3^+^ T-cell, CD3^+^CD8^-^ T-cell, CD3^+^FoxP3^+^ regulatory T-cell and CD8^+^Ki67^+^ T-cell density is significantly associated with positive HER2 status (Wilcoxon-Mann-Whitney test). Higher intraepithelial CD3^+^FoxP3^+^ T-cell and CD20^+^ B-cell density was associated with Ki67 ≥14%, while higher CD68^+^ cell density was associated with Ki67 <14%.

Significant associations are indicated as:

*: *P* < .05, **: *P* < .01.

The central line in boxes represent the median value, boundaries of boxes represent the interquartile range (IQR), and ends of whiskers represent values at 1.5 x IQR.

^a^: outcome, 0 = controls and 1 = cases

^b^: histologic grade was based on nuclear grade

^c^: periductal fibrosis, 0 = absent, 1 = present

^d^: ER, 0 = negative, 1 = positive; ER was considered positive when ≥ 10% of the luminal epithelial cells showed nuclear staining of any intensity

^e^: HER2, 0 = negative, 1= positive

^f^: Ki67 expression in DCIS cells, 0 = <14%, 1 = ≥14%

^g^: COX-2, 0 = low expression in DCIS cells, 1 = high expression in DCIS cells

**
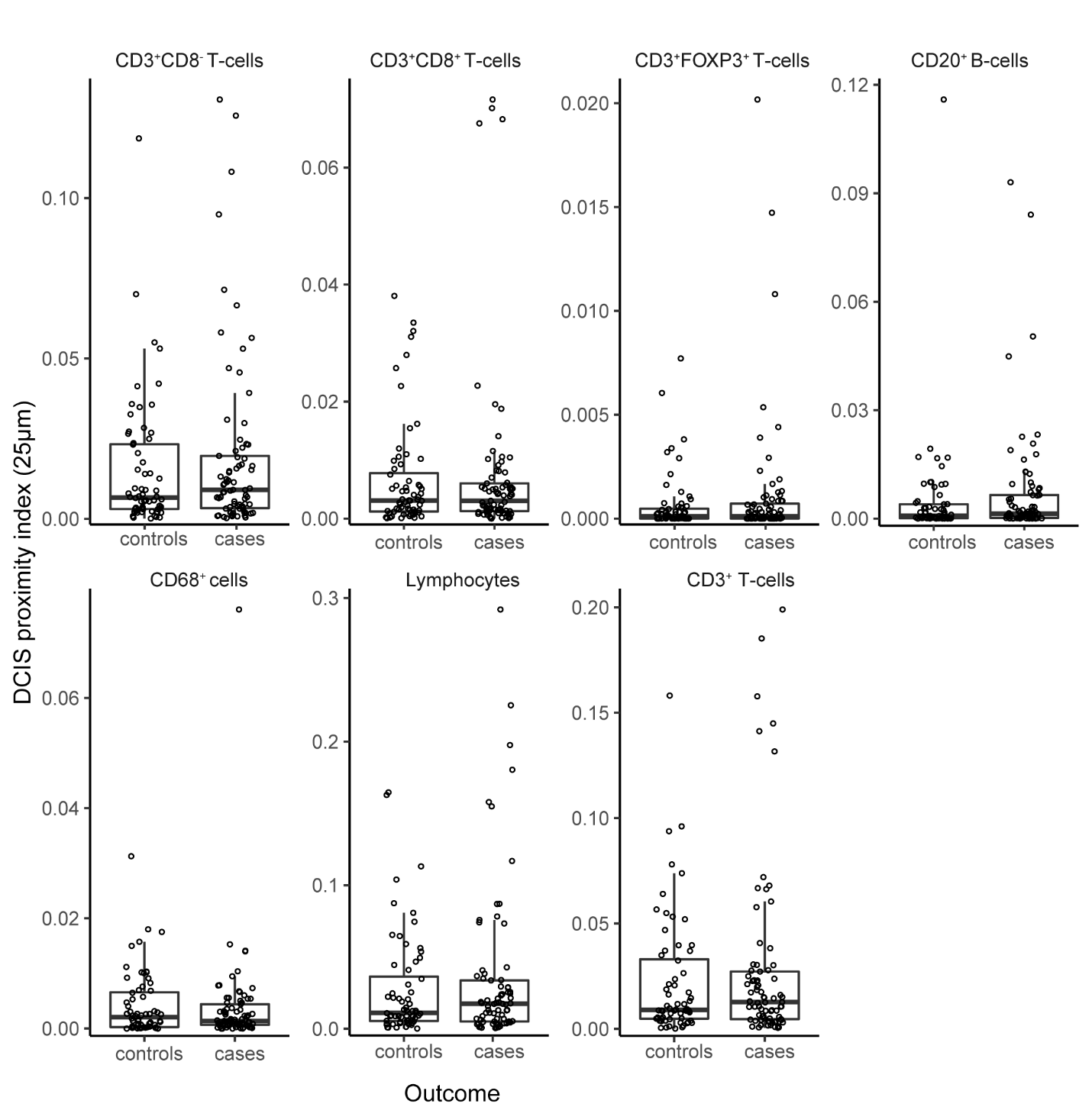
**

**Supplementary figure 2. DCIS proximity index**

The DCIS proximity index (average number of immune cells to closest DCIS cell within 25 µm) does not differ between cases and controls (*P* > .05, Wilcoxon-Mann-Whitney test). The central line in boxes represent the median value, boundaries of boxes represent the interquartile range (IQR), and ends of whiskers represent values at 1.5 x IQR.

**
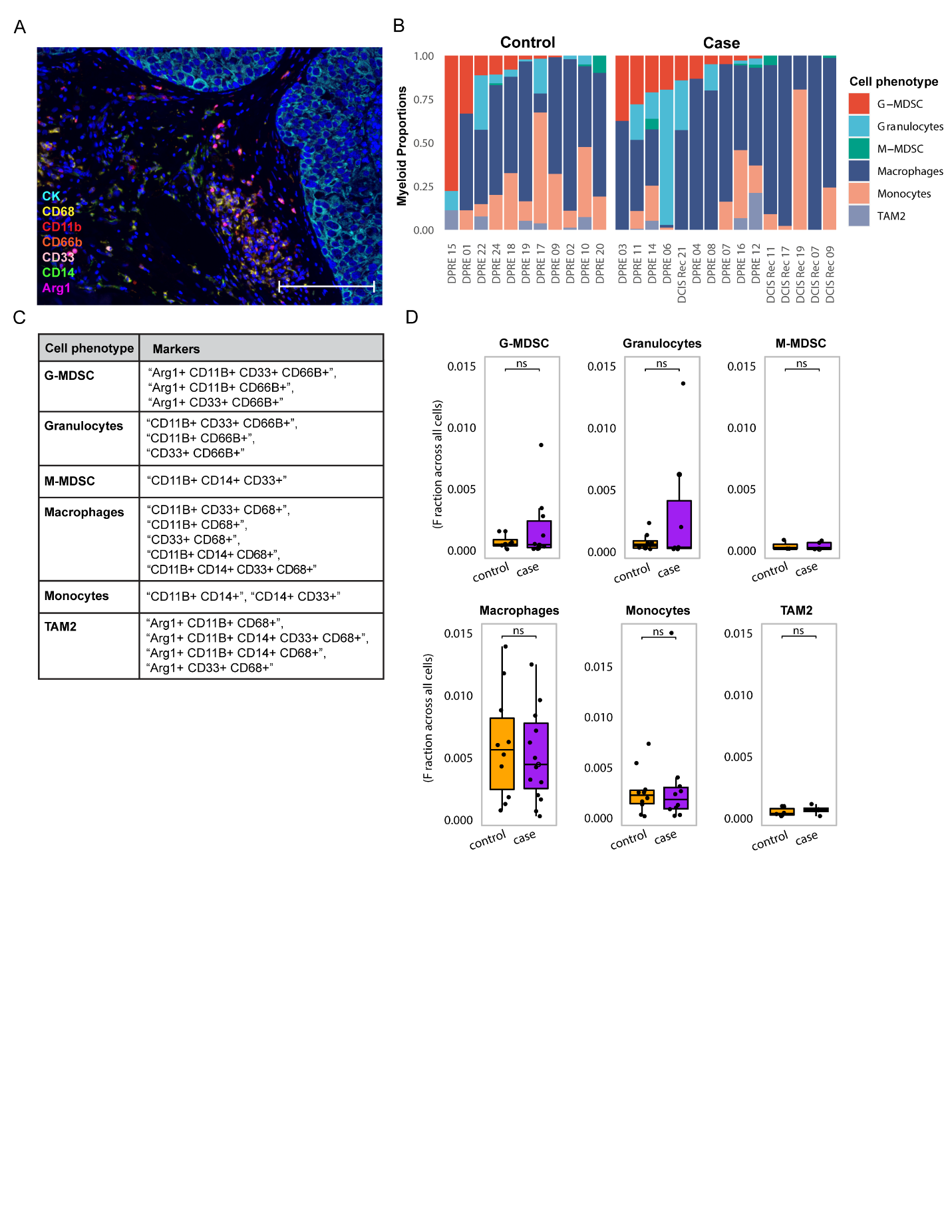
Supplementary figure 3.**

Multiplex immunofluorescence for myeloid cell profiling. A. Staining of a primary DCIS with invasive recurrence sample using a myeloid marker panel by pseudo coloring (cyan, CK; yellow, CD68; red, CD11b; orange, CD66b; pink, CD33; green, CD14; magenta, Arg1) using representative 7-color multiplex immunofluorescence. The scalebar represents 50 µm. B. Proportion of myeloid cell phenotypes colored by different myeloid cells grouped by controls and cases. C. Table showing myeloid lineage markers and corresponding cell phenotypes. D. Boxplots comparing the frequency of myeloid cell phenotypes as a proportion of total number of cells profiled including tumour and other stromal cells between controls and cases (Wilcoxon test). The central line in boxes represent the median value, boundaries of boxes represent the interquartile range (IQR), and ends of whiskers represent values at 1.5 x IQR.

**Supplementary table 1** Antibodies and reagents of multiplex immunofluorescent (IF) imaging

A.

| **Antigen** | **Clone** | **Vendor** | **Cat. No.** | **Dilution** |
| --- | --- | --- | --- | --- |
| CD3 | \| SP7 \| \| --- \| | \| SpringBio \| \| --- \| | \| M3074 \| \| --- \| | 1/400 |
| CD8 | \| C8/144B \| \| --- \| | DAKO | \| M7103 \| \| --- \| | 1/100 |
| CD20 | \| L26 \| \| --- \| | DAKO | \| M0755 \| \| --- \| | 1/500 |
| FoxP3 | \| 236A/47 \| \| --- \| | \| AbCam \| \| --- \| | \| AB20034 \| \| --- \| | 1/50 |
| CD68 | \| KP1 \| \| --- \| | \| Dako \| \| --- \| | \| M0814 \| \| --- \| | 1/500 |
| pancytokeratin | \| AE1AE3 \| \| --- \| | \| Thermo Sci \| \| --- \| | \| MS-343P \| \| --- \| | 1/250 |

B.

| **Reagent** | **Vendor** | **Cat. No.** | **Dilution** |
| --- | --- | --- | --- |
| Discovery inhibitor | Ventana | 760-4840 | RTU |
| Opal Polymer HRP | Perkin Elmer | ARH1001EA | RTU |
| Amplification Diluent | Perkin Elmer | FP1498 | RTU |
| Antibody diluent/block | Perkin Elmer | ARD1001EA | RTU |
| Fluoromount-G | Southern Biotech. | 0100-01 | RTU |
| Opal 520 | Perkin Elmer | FP1487A | 1/75 |
| Opal 540 | Perkin Elmer | FP1494A | 1/75 |
| Opal 570 | Perkin Elmer | FP1488A | 1/200 |
| Opal 620 | Perkin Elmer | FP1495A | 1/75 |
| Opal 650 | Perkin Elmer | FP1496A | 1/75 |
| Opal 690 | Perkin Elmer | FP1497A | 1/50 |
| DAPI | Perkin Elmer | FP1490 | 1/25 |

C.

| **Antigen** | **Species** | **Clone** | **Vendor** | **Cat. No.** |
| --- | --- | --- | --- | --- |
| CD66b | Mousemab | G10F5 (M) | BioLegend | 305102 |
| CD14 | Rmab | SP192 | Abcam | ab183322 |
| CD33 | Mousemab | PWS44 (M) | Leica Biosystems | NCL-L-CD33 |
| Arginase 1 | Rmab | D4E3M (R) | Cell Signaling | #93668S |
| CD68 | Mouseab | PG-M1 (M) | DAKO | M087601-2 |
| CD11b | Rmab | EPR1344 (R) | Abcam | ab133357 |
| pancytokeratin | Mousemab | AE1/AE3 | DAKO | M351501-2 |

RTU = ready to use.

A. Antibodies and B. reagents for immunofluorescence (IF) multispectral imaging of B-cells, T-cells and CD68^+^ cells. C. Antibodies for multiplex IF of myeloid cells.

**Supplementary table 2. Postprocessing corrections multiplex imaging**

| Cell with observed markers | Cell phenotyped |
| --- | --- |
| CD3 and CD20 double positivity | according to marker with highest intensity |
| FOXP3, CD8 and CD3 positivity | as a CD8-positive T-cell |
| CD68 and pankeratin double positivity | according to the highest intensity |
| Single positivity for CD8 | as a CD8-positive T-cell |
| CD8 and CD20 double positivity | as a CD20-positive B-cell |
| CD3, CD8 and CD20 positivity | according to highest intensity of CD8 and CD20 |

**Supplementary table 3.** Median stromal immune cell density in relation to clinical, histopathological and immunohistochemical characteristics of female primary DCIS patients treated with breast conserving surgery alone, who subsequently did (cases) or did not (controls) develop subsequent ipsilateral invasive breast cancer.

| **Characteristics^c^** | n | Lymphocytes/mm^2^  *P*-value^a^ | CD3^+^ T-cells/mm^2^  *P*-value^a^ | CD20^+^ B-cells/mm^2^  *P*-value^a^ | CD3^+^CD8^-^ T-cells/  mm^2^  *P*-value^a^ | CD3^+^CD8^+^ T-cells/mm^2^  *P*-value^a^ | CD3^+^FOXP3^+^ T-cells/mm^2^  *P*-value | CD68^+^ cells/mm^2^  *P*-value | CD8^+^Ki67^+^ T-cells/mm^2^  *P*-value |
| --- | --- | --- | --- | --- | --- | --- | --- | --- | --- |
| **Clinical characteristics** |  |  |  |  |  |  |  |  |  |
| All patients | 141 | 370 | 235 | 50.7 | 178 | 54.7 | 3.67 | 17.8 | 0.97 |
| Outcome |  | .62 | .57 | .51 | .62 | .58 | .51 | .14 | .53 |
| No iIBC (controls) | 64 | 380 | 232 | 47.8 | 179 | 53.6 | 4.01 | 24.3 | 0.95 |
| iIBC (cases) | 77 | 366 | 264 | 58.2 | 178 | 56.7 | 3.58 | 16.8 | 0.99 |
| Age (years) |  | .97 | .97 | .70 | .87 | .78 | .64 | .50 | .43 |
| ≤ 50 years | 28 | 370 | 278 | 50.6 | 202 | 61.5 | 2.45 | 20.4 | 1.60 |
| > 50 years | 113 | 370 | 233 | 54.4 | 173 | 54.3 | 3.80 | 17.8 | 0.95 |
| Clinical presentation of DCIS |  | .18 | .23 | .26 | .31 | .31 | .088 | .46 | .15 |
| Screen-detected | 69 | 201 | 176 | 23.5 | 144 | 40.2 | 1.33 | 12.8 | 0.39 |
| Non-screen-related | 8 | 424 | 315 | 62.4 | 230 | 54.3 | 5.35 | 20.6 | 1.00 |
| Unknown^b^ | 64 | 296 | 219 | 47.8 | 176 | 56.9 | 3.00 | 14.3 | 1.06 |
| Time to iIBC^d^ |  | .26 | .19 | .30 | .19 | .26 | .078 | .43 | .24 |
| ≤5.4 years | 38 | 439 | 320 | 70.6 | 240 | 62.0 | 4.25 | 18.3 | 1.57 |
| >5.4 years | 39 | 214 | 183 | 33.9 | 143 | 40.9 | 1.67 | 17.1 | 0.81 |
| **Histopathology** |  |  |  |  |  |  |  |  |  |
| Lesion size |  | .69 | .69 | .37 | .68 | .69 | .78 | .72 | .42 |
| ≤10 mm | 29 | 314 | 232 | 31.1 | 180 | 59.7 | 2.46 | 12.8 | 0.94 |
| >10 mm | 24 | 355 | 269 | 57.3 | 204 | 57.6 | 3.81 | 16.6 | 1.32 |
| Unknown^b^ | 88 | 393 | 219 | 49.4 | 173 | 51.7 | 3.74 | 18.8 | 0.94 |
| Margin Status |  | .49 | .54 | .42 | .46 | .90 | .13 | .20 | .14 |
| Free | 69 | 314 | 263 | 33.9 | 178 | 53.0 | 2.50 | 14.1 | 0.83 |
| Not free | 46 | 439 | 303 | 60.1 | 238 | 60.3 | 4.20 | 31.7 | 1.36 |
| Unknown^b^ | 26 | 339 | 209 | 59.8 | 163 | 55.4 | 4.00 | 17.0 | 1.54 |
| Dominant growth pattern |  | .082 | .063 | .12 | **.048** | .14 | **.033** | .45 | .70 |
| Clinging | 6 | 312 | 192 | 53.9 | 133 | 59.0 | 2.25 | 11.0 | 1.44 |
| (Micro-)papillary | 16 | 245 | 187 | 49.2 | 161 | 49.7 | 3.98 | 21.7 | 1.24 |
| Cribriform | 30 | 237 | 199 | 34.1 | 160 | 40.9 | 1.54 | 12.6 | 0.94 |
| Solid | 89 | 433 | 321 | 64.9 | 244 | 63.5 | 4.10 | 20.5 | 0.99 |
| Histologic grade |  | **< .001** | **< .001** | **< .001** | **< .001** | **.004** | **.043** | .34 | .061 |
| Grade 1 | 17 | 314 | 182 | 34.3 | 111 | 44.0 | 1.68 | 14.3 | 1.43 |
| Grade 2 | 91 | 254 | 207 | 32.3 | 158 | 51.1 | 3.09 | 16.8 | 0.84 |
| Grade 3 | 33 | 756 | 462 | 197 | 397 | 97.1 | 5.35 | 29.8 | 1.56 |
| Comedonecrosis |  | **.005** | **.006** | **.003** | **.003** | .085 | **.022** | 0.54 | 0.64 |
| Absent | 29 | 192 | 164 | 15.8 | 116 | 42.6 | 2.04 | 13.6 | 1.12 |
| Present | 112 | 421 | 289 | 60.1 | 219 | 59.6 | 4.03 | 19.0 | 0.95 |
| Calcifications |  | .32 | .33 | .73 | .38 | .27 | .33 | .27 | .83 |
| Absent | 24 | 198 | 180 | 47.8 | 143 | 53.8 | 2.06 | 27.0 | 0.81 |
| Present | 117 | 390 | 268 | 54.7 | 191 | 55.9 | 3.95 | 17.1 | 0.99 |
| Periductal fibrosis |  | **.016** | **.011** | **.017** | **.012** | **.031** | **.016** | **<.001** | **.002** |
| Absent | 99 | 284 | 211 | 37.3 | 158 | 47.4 | 3.18 | 12.4 | 0.73 |
| Present | 42 | 450 | 361 | 75.6 | 260 | 68.5 | 7.86 | 43.6 | 1.78 |
| **IHC** |  |  |  |  |  |  |  |  |  |
| ER^e^ |  | **<.001** | **<.001** | **<.001** | **<.001** | **.013** | **.022** | **.006** | **.004** |
| Negative | 28 | 998 | 649 | 277 | 479 | 101 | 6.26 | 44.6 | 2.59 |
| Positive | 112 | 261 | 205 | 32.2 | 156 | 52.1 | 3.20 | 14.3 | 0.87 |
| N/A | 1 |  |  |  |  |  |  |  |  |
| PR^e^ |  | **<.001** | **<.001** | **<.001** | **<.001** | **<.001** | **<.001** | **.029** | **.006** |
| Negative | 53 | 606 | 447 | 194 | 322 | 97.1 | 5.98 | 27.5 | 1.66 |
| Positive | 87 | 230 | 183 | 28.7 | 141 | 44.0 | 2.50 | 14.1 | 0.82 |
| N/A | 1 |  |  |  |  |  |  |  |  |
| HER2 |  | **<.001** | **< .001** | **< .001** | **< .001** | **< .001** | **.002** | **.034** | **< .001** |
| Negative | 90 | 235 | 187 | 31.6 | 146 | 44.4 | 2.63 | 14.8 | 0.81 |
| Positive | 39 | 760 | 462 | 244 | 397 | 97.1 | 4.57 | 29.8 | 1.92 |
| N/A | 2 |  |  |  |  |  |  |  |  |
| COX-2 |  | .76 | .99 | .20 | .90 | .68 | .29 | .40 | .24 |
| Low | 12 | 355 | 298 | 35.3 | 211 | 68.8 | 3.09 | 11.5 | 0.39 |
| High | 128 | 380 | 247 | 50.6 | 179 | 53.6 | 3.81 | 19.0 | 1.00 |
| N/A | 1 |  |  |  |  |  |  |  |  |
| Ki67 |  | **.001** | **.004** | **<.001** | **.004** | .071 | **<.001** | .60 | **.007** |
| < 14% | 103 | 313 | 221 | 41.8 | 168 | 54.3 | 3.50 | 18.7 | 0.89 |
| ≥ 14% | 15 | 736 | 462 | 271 | 328 | 126 | 17.1 | 27.5 | 4.16 |
| N/A | 23 | 194 | 178 | 25.2 | 116 | 49.7 | 2.04 | 13.1 | N/A |

N/A: Not assessable; N/As were not included in the analysis.

HR: hormone receptor; HR+ = ER positive and/or PR positive; HR- = ER negative and PR negative.

^a^: Comparisons were made with the Wilcoxon-Mann-Whitney test or the Kruskal-Wallis test. Nominal P-values were calculated with permutation.

^b^: data in the “unknown” category were not included in the analysis

^c^: stromal lymphocyte, CD3^+^ T-cell, CD3^+^CD8^+^ T-cell, CD20^+^ B-cell, and CD68^+^ cell density was assessable in 141 DCIS patients. Stromal CD3^+^FOXP3^+^ T-cell density was assessable in 127 patients and CD8^+^Ki67^+^ T-cell density was assessable in 118 DCIS patients.

^d^: time to iIBC is only applicable to cases.

^e^: ER and PR were considered positive when ≥10% of luminal epithelial cells showed nuclear staining of any intensity.

**Supplementary table 4.** Median intraepithelial DCIS immune cell density in female primary DCIS patients treated with breast conserving surgery alone, who subsequently did (cases) or did not (controls) develop subsequent ipsilateral invasive breast cancer.

|  | n | Lymphocytes/mm^2^  *P*-value^a^ | CD3^+^ T-cells/mm^2^  *P*-value^a^ | CD20^+^ B-cells/mm^2^  *P*-value^a^ | CD3^+^CD8^-^ T-cells/  mm^2^  *P*-value^a^ | CD3^+^CD8^+^ T-cells/mm^2^  *P*-value^a^ | CD3^+^FOXP3^+^ T-cells/mm^2^  *P*-value^a^ | CD68^+^ cells/mm^2^  *P*-value^a^ | CD8^+^Ki67^+^ T-cells/mm^2^  *P*-value^a^ |
| --- | --- | --- | --- | --- | --- | --- | --- | --- | --- |
| All patients | 141 | 10.1 | 10.1 | 0 | 6.72 | 2.16 | 0.25^b^ | 20.3 | 0.85^b^ |
| Outcome |  | 1 | 1 | 1 | 1 | 1 | 1 | 1 | 1 |
| No iIBC (controls) | 64 | 9.85 | 9.62 | 0 | 6.67 | 2.44 | 0.27 | 19.1 | 0.74 |
| iIBC (cases) | 77 | 10.3 | 10.3 | 0 | 6.82 | 1.87 | 0.24 | 21.4 | 1.12 |

^a^: Comparisons were made with the Wilcoxon-Mann-Whitney test. Nominal *P*-values were calculated with permutation.

^b^: CD3^+^FOXP3^+^ T-cell density was assessable in 127 patients and CD8^+^Ki67^+^ T-cell density was assessable in 118 DCIS patients.

**Supplementary table 5.** Stromal immune cell density in relation to DCIS subtype.

|  | n(%) | | Lymphocytes  /mm^2^  *P*-value | CD3^+^ T-cells  /mm^2^  *P*-value | CD20^+^ B-cells/mm^2^  *P*-value | CD3^+^CD8^-^ T-cells  /mm^2^  *P*-value | CD3^+^CD8^+^ T-cells/mm^2^  *P*-value | CD3^+^FOXP3^+^ T-cells/mm^2^  *P*-value | CD68^+^ cells/mm^2^  *P*-value | CD8^+^Ki67^+^ T-cells/mm^2^  *P*-value |
| --- | --- | --- | --- | --- | --- | --- | --- | --- | --- | --- |
| subtype |  |  | <.001 | <.001 | <.001 | <.001 | .001 | .009 | .049 | .005 |
| ER+HER2- | 95 | (67) | 230 | 182 | 29.3 | 141 | 44.3 | 2.74 | 14.1 | 0.82 |
| ER+HER2+ | 16 | (11) | 508 | 377 | 110 | 247 | 103 | 4.20 | 23.6 | 1.43 |
| ER-HER2+ | 23 | (16) | 1086 | 745 | 285 | 483 | 97.1 | 16.5 | 43.6 | 2.85 |
| ER-HER2- | 5 | (4) | 486 | 426 | 185 | 322 | 105 | 0.92 | 59.1 | 0.19 |
| N/A | 2 | (1) |  |  |  |  |  |  |  |  |

Median stromal immune cell density for each immunohistochemical DCIS subtype. Comparisons were made with the Kruskal-Wallis test.

**Supplementary table 6.** Median stromal and intraepithelial immune cell ratios in cases and controls

| Immune cell ratio | Stroma | | | | | DCIS epithelium | | | | |
| --- | --- | --- | --- | --- | --- | --- | --- | --- | --- | --- |
|  | All patients (n) | Cases (n) | Controls (n) | Nominal *P*-value | FDR | All patients^a^ (n) | Cases^a^ (n) | Controls^a^ (n) | Nominal *P*-value | FDR |
| CD3^+^ T-cells/lymphocytes | 0.84 (141) | 0.81 (77) | 0.87 (64) | 0.257 | 0.426 | 1.00 (140) | 1.00 (76) | 1.00 (64) | 0.881 | 0.968 |
| CD20^+^ B-cells/lymphocytes | 0.16 (141) | 0.19 (77) | 0.13 (64) | 0.257 | 0.426 | 0.00 (140) | 0.00 (76) | 0.00 (64) | 0.883 | 0.968 |
| CD3^+^CD8^-^ T-cells/lymphocytes | 0.59 (141) | 0.58 (77) | 0.60 (64) | 0.842 | 0.842 | 0.70 (140) | 0.73 (76) | 0.67 (64) | 0.244 | 0.896 |
| CD3^+^CD8^+^ T-cells/lymphocytes | 0.19 (141) | 0.17 (77) | 0.20 (64) | 0.174 | 0.426 | 0.23 (140) | 0.18 (76) | 0.25 (64) | 0.098 | 0.606 |
| CD3^+^FOXP3^+^ T-cells/lymphocytes | 0.012 (127) | 0.011 (71) | 0.014 (56) | 0.437 | 0.582 | 0.019 (126) | 0.018 (70) | 0.019 (56) | 0.897 | 0.968 |
| CD68^+^ macrophages/lymphocytes | 0.049 (141) | 0.041 (77) | 0.053 (64) | 0.202 | 0.426 | 1.79 (141) | 1.86 (77) | 1.77 (64) | 0.606 | 0.968 |
| CD8^+^Ki67^+^ T-cells/lymphocytes | 0.003 (118) | 0.003 (64) | 0.003 (54) | 0.143 | 0.426 | 0.091 (118) | 0.11 (64) | 0.087 (54) | 0.310 | 0.896 |
| CD20^+^/CD3^+^ T-cells | 0.19 (141) | 0.24 (77) | 0.15 (64) | 0.257 | 0.426 | 0.00 (140) | 0.00 (76) | 0.00 (64) | 0.882 | 0.968 |
| CD3^+^CD8^-^ T-cells/CD3^+^ T-cells | 0.74 (141) | 0.75 (77) | 0.73 (64) | 0.283 | 0.426 | 0.72 (140) | 0.74 (76) | 0.69 (64) | 0.191 | 0.892 |
| CD3^+^CD8^+^ T-cells/CD3^+^ T-cells | 0.24 (141) | 0.23 (77) | 0.24 (64) | 0.299 | 0.426 | 0.23 (140) | 0.18 (76) | 0.26 (64) | 0.106 | 0.606 |
| CD3^+^FOXP3^+^ T-cells/CD3^+^ T-cells | 0.015 (127) | 0.015 (71) | 0.016 (56) | 0.537 | 0.658 | 0.019 (126) | 0.019 (70) | 0.019 (56) | 0.903 | 0.968 |
| CD68^+^ macrophages/CD3^+^ T-cells | 0.066 (141) | 0.053 (77) | 0.070 (64) | 0.211 | 0.426 | 1.80 (141) | 1.86 (77) | 1.77 (64) | 0.561 | 0.968 |
| CD8^+^Ki67^+^ T-cells/CD3^+^ T-cells | 0.004 (118) | 0.004 (64) | 0.003 (54) | 0.137 | 0.426 | 0.094 (118) | 0.11 (64) | 0.091 (54) | 0.315 | 0.896 |
| CD3^+^CD8^-^ T-cells/CD20^+^ B-cells | 3.65 (141) | 2.84 (77) | 4.61 (64) | 0.276 | 0.426 | Inf^b^ (139) | Inf^b^ (75) | Inf^b^ (64) | 0.945 | 0.968 |
| CD3^+^CD8^+^ T-cells/CD20^+^ B-cells | 1.30 (141) | 1.06 (77) | 1.55 (64) | 0.185 | 0.426 | Inf^b^ (139) | Inf^b^ (72) | Inf^b^ (62) | 0.622 | 0.968 |
| CD3^+^FOXP3^+^ T-cells/CD20^+^ B-cells | 0.074 (127) | 0.055 (71) | 0.11 (56) | 0.111 | 0.426 | 7.56 (94) | 9.00 (53) | 7.17 (41) | 0.959 | 0.968 |
| CD68^+^ macrophages/CD20^+^ B-cells | 0.39 (141) | 0.30 (77) | 0.46 (64) | 0.075 | 0.426 | Inf^b^ (140) | Inf^b^ (76) | Inf^b^ (64) | 0.791 | 0.968 |
| CD8^+^Ki67^+^ T-cells/CD20^+^ B-cells | 0.020 (118) | 0.019 (64) | 0.023 (54) | 0.634 | 0.658 | Inf^b^ (101) | Inf^b^ (57) | 345 (44) | 0.511 | 0.968 |
| CD3^+^CD8^+^ T-cells/CD3^+^CD8^-^ T-cells | 0.32 (141) | 0.30 (77) | 0.33 (64) | 0.304 | 0.426 | 0.32 (140) | 0.23 (76) | 0.37 (64) | 0.108 | 0.606 |
| CD3^+^FOXP3^+^ T-cells/CD3^+^CD8^-^ T-cells | 0.021 (127) | 0.020 (71) | 0.022 (56) | 0.563 | 0.658 | 0.027 (125) | 0.027 (69) | 0.028 (56) | 0.739 | 0.968 |
| CD68^+^ macrophages/CD3^+^CD8^-^ T-cells | 0.089 (141) | 0.075 (77) | 0.099 (64) | 0.139 | 0.426 | 2.59 (141) | 2.59 (77) | 2.61 (64) | 0.864 | 0.968 |
| CD8^+^Ki67^+^ T-cells/CD3^+^CD8^-^ T-cells | 0.005 (118) | 0.005 (64) | 0.004 (54) | 0.208 | 0.426 | 0.14 (117) | 0.14 (63) | 0.14 (54) | 0.352 | 0.896 |
| CD3^+^FOXP3^+^ T-cells/CD3^+^CD8^+^ T-cells | 0.058 (127) | 0.055 (71) | 0.061 (56) | 0.634 | 0.658 | 0.088 (120) | 0.088 (66) | 0.092 (54) | 0.693 | 0.968 |
| CD68^+^ macrophages/CD3^+^CD8^+^ T-cells | 0.29 (141) | 0.30 (77) | 0.29 (64) | 0.613 | 0.658 | 10.1 (141) | 14.3 (77) | 7.41 (64) | 0.101 | 0.606 |
| CD8^+^Ki67^+^ T-cells/CD3^+^CD8^+^ T-cells | 0.019 (118) | 0.024 (64) | 0.014 (54) | 0.066 | 0.426 | 0.37 (114) | 0.53 (61) | 0.34 (53) | 0.108 | 0.606 |
| CD68^+^ macrophages/CD3^+^FOXP3^+^ T-cells | 4.60 (127) | 4.39 (71) | 4.72 (56) | 0.581 | 0.658 | 131 (127) | 161 (71) | 123 (56) | 0.968 | 0.968 |
| CD8^+^Ki67^+^ T-cells/CD3^+^FOXP3^+^ T-cells | 0.24 (118) | 0.35 (57) | 0.20 (44) | 0.118 | 0.426 | 5.34 (95) | 7.25 (52) | 3.63 (43) | 0.328 | 0.896 |
| CD8^+^Ki67^+^ T-cells/CD68^+^ | 0.048 (118) | 0.053 (64) | 0.032 (54) | **0.043** | 0.426 | 0.057 (117) | 0.058 (53) | 0.044 (64) | 0.746 | 0.968 |

Comparisons were made with the Wilcoxon-Mann-Whitney test. Nominal *P*-values were calculated with permutation. Multiple testing correction was performed using the FDR.

^a^: immune cell ratio was not assessable in patients of which both immune cell subsets were “0”.

^b^: Inf: infinitive

**Supplementary table 7.** Median stromal immune cell density in the ER^+^/HER2^-^ DCIS subgroup and the periductal fibrosis-absent subgroup

| Median immune cell density (cells/mm^2^) | Stroma | | | | | DCIS epithelium | | | | |
| --- | --- | --- | --- | --- | --- | --- | --- | --- | --- | --- |
|  | All patients (n) | Cases (n) | Controls (n) | Nominal *P*-value | FDR | All patients (n) | Cases (n) | Controls (n) | Nominal *P*-value | FDR |
| **ER+/HER2- subgroup** | | | | | | | | | | |
| Lymphocytes | 230 (95) | 204 (53) | 250 (42) | 0.423 | 0.676 | 8.96 (95) | 8.96 (53) | 9.16 (42) | 0.911 | 0.911 |
| CD3^+^ T-cells | 182 (95) | 182 (53) | 204 (42) | 0.809 | 0.676 | 8.81 (95) | 8.88 (53) | 8.80 (42) | 0.887 | 0.911 |
| CD20^+^ B-cells | 29.3 (95) | 29.3 (53) | 29.8 (42) | 0.367 | 0.818 | 0.00 (95) | 0.11 (53) | 0.18 (42) | 0.391 | 0.911 |
| CD3^+^CD8^-^ T-cells | 141 (95) | 128 (53) | 152 (42) | 0.330 | 0.676 | 6.04 (95) | 6.30 (53) | 5.58 (42) | 0.759 | 0.911 |
| CD3^+^CD8^+^ T-cells | 44.3 (95) | 45.0 (53) | 42.6 (42) | 0.247 | 0.818 | 1.87 (95) | 1.77 (53) | 2.02 (42) | 0.750 | 0.911 |
| CD3^+^FOXP3^+^ T-cells | 2.74 (84) | 1.42 (47) | 4.25 (37) | **0.034** | 0.272 | 0.16 (84) | 0.053 (47) | 0.25 (37) | 0.270 | 0.911 |
| CD68^+^ cells | 14.1 (95) | 12.8 (53) | 15.8 (42) | 0.197 | 0.676 | 21.4 (95) | 21.7 (53) | 19.4 (42) | 0.298 | 0.911 |
| CD8^+^Ki67^+^ T-cells | 0.82 (76) | 0.22 (42) | 0.31 (34) | 0.818 | 0.818 | 0.67 (76) | 0.71 (42) | 0.62 (34) | 0.519 | 0.911 |
| **Periductal fibrosis absent subgroup** | | | | | | | | | | |
| Lymphocytes | 284 (98) | 259 (55) | 314 (43) | 0.816 | 0.816 | 10.1 (98) | 11.6 (55) | 9.83 (43) | 0.609 | 0.696 |
| CD3^+^ T-cells | 211 (98) | 195 (55) | 235 (43) | 0.675 | 0.816 | 10.1 (98) | 11.6 (55) | 9.27 (43) | 0.594 | 0.696 |
| CD20^+^ B-cells | 37.3 (98) | 41.3 (55) | 28.7 (43) | 0.396 | 0.816 | 0.00 (98) | 0.00 (55) | 0.037 (43) | 0.495 | 0.696 |
| CD3^+^CD8^-^ T-cells | 158 (98) | 143 (55) | 162 (43) | 0.794 | 0.816 | 6.72 (98) | 6.82 (55) | 6.72 (43) | 0.318 | 0.696 |
| CD3^+^CD8^+^ T-cells | 47.4 (98) | 44.3 (55) | 52.9 (43) | 0.569 | 0.816 | 2.00 (98) | 1.87 (55) | 2.03 (43) | 0.728 | 0.728 |
| CD3^+^FOXP3^+^ T-cells | 3.18 (98) | 3.01 (51) | 3.80 (37) | 0.358 | 0.816 | 0.23 (88) | 0.18 (51) | 0.25 (37) | 0.664 | 0.696 |
| CD68^+^ cells | 12.4 (98) | 11.2 (55) | 14.3 (43) | 0.281 | 0.816 | 18.5 (98) | 16.0 (55) | 21.0 (43) | 0.238 | 0.696 |
| CD8^+^Ki67^+^ T-cells | 0.73 (81) | 0.73 (45) | 0.69 (36) | 0.512 | 0.816 | 0.75 (81) | 1.09 (45) | 0.47 (36) | 0.183 | 0.696 |

Comparisons were made with the Wilcoxon-Mann-Whitney test. Nominal *P*-values were calculated with permutation. Multiple testing correction was performed using the FDR.

**Supplementary table 8.** Characteristics of DCIS patients for myeloid multiplex IF

| **Sample** | **Patient cohort^a^** | **iIBC^b^** | **Age at diagnosis** | **DCIS grade** | **Type of surgery^c^** | **Radiotherapy^d^** | **Endocrine therapy^d^** |
| --- | --- | --- | --- | --- | --- | --- | --- |
| DPRE01 | D | 0 | 48 | 2-Intermediate | BCS | 0 | 0 |
| DPRE02 | D | 0 | 64 | 2-Intermediate | BCS | 0 | 0 |
| DPRE03 | D | 1 | 56 | 3-High | BCS | 0 | 0 |
| DPRE04 | D | 1 | 46 | 3-High | BCS | 0 | 0 |
| DPRE06 | D | 1 | 56 | 1-Low | BCS | 0 | 0 |
| DPRE07 | D | 1 | 43 | 1-Low | BCS | 0 | 0 |
| DPRE08 | D | 1 | 67 | 3-High | BCS | 0 | 0 |
| DPRE09 | D | 0 | 44 | 2-Intermediate | BCS | 0 | 0 |
| DPRE10 | D | 0 | 81 | 2-Intermediate | BCS | 0 | 0 |
| DPRE11 | D | 1 | 66 | 3-High | BCS | 0 | 0 |
| DPRE12 | D | 1 | 68 | 2-Intermediate | BCS | 0 | 0 |
| DPRE14 | D | 1 | 65 | 2-Intermediate | BCS | 0 | 0 |
| DPRE15 | D | 0 | 63 | 2-Intermediate | BCS | 0 | 0 |
| DPRE16 | D | 1 | 67 | 3-High | BCS | 0 | 0 |
| DPRE17 | D | 0 | 62 | 3-High | BCS | 0 | 0 |
| DPRE18 | D | 0 | 56 | 3-High | BCS | 0 | 0 |
| DPRE19 | D | 0 | 56 | 3-High | BCS | 0 | 0 |
| DPRE20 | D | 0 | 60 | 3-High | BCS | 0 | 0 |
| DPRE22 | M | 0 | 58 | 3-High | Bilateral Mastectomy | unknown | unknown |
| DPRE24 | M | 0 | 52 | 2-Intermediate | Unilateral Mastectomy | unknown | unknown |
| DCIS Rec 07 | M | 1 | 49 | 3-High | Segmental Mastectomy | 0 | unknown |
| DCIS Rec 09 | M | 1 | 50 | 3-High | Segmental Mastectomy | 1 | 1 |
| DCIS Rec 11 | M | 1 | 55 | 2-Intermediate | Segmental Mastectomy | 0 | unknown |
| DCIS Rec 17 | M | 1 | 44 | 2-Intermediate | Segmental Mastectomy | 0 | unknown |
| DCIS Rec 19 | M | 1 | 41 | 3-High | Segmental Mastectomy | 1 | 0 |
| DCIS Rec 21 | M | 1 | 54 | 2-Intermediate | Segmental Mastectomy | 1 | 0 |

^a^: Patient cohort: D=nation-wide Dutch patient; M: patient from MD Anderson Cancer Center

^b^: iIBC: subsequent ipsilateral invasive breast cancer, 0 = no iIBC, 1= subsequent iIBC

^c^: BCS: breast conserving surgery

^d^: Radiotherapy, endocrine therapy: 0= no, 1= yes

**Supplementary table 9.** Concordance of DCIS TILs score on different H&E slides

| DCIS patient | DCIS TIL score (%) | | | |
| --- | --- | --- | --- | --- |
|  | Slide 1 | Slide 2 | Slide 3^a^ | Slide 4^a^ |
| 1 | 1 | 5 | 5 | NA |
| 2 | 5 | 5 | NA | NA |
| 3 | 10 | 10 | NA | NA |
| 4 | 5 | 1 | 5 | NA |
| 5 | 5 | 5 | 5 | NA |
| 6 | 25 | 20 | 20 | 30 |
| 7 | 5 | 1 | NA | NA |
| 8 | 5 | 10 | 5 | NA |
| 9 | 10 | 10 | 5 | 5 |
| 10 | 5 | 10 | NA | NA |
| 11 | 5 | 5 | 5 | 5 |
| 12 | 20 | 30 | 20 | NA |
| 13 | 20 | 30 | 20 | NA |
| 14 | 10 | 5 | 1 | NA |
| 15 | 1 | 0 | 0 | NA |
| 16 | 10 | 5 | 5 | NA |
| 17 | 10 | 5 | 5 | NA |
| 18 | 20 | 30 | 20 | NA |
| 19 | 1 | 1 | NA | NA |
| 20 | 20 | 20 | 20 | NA |

Concordance of DCIS TILs score on different H&E slides of the same patient, scored by one breast pathologist in a subset of 20 DCIS patients treated with BCS alone of the Dutch DCIS series.

^a^: TIL scores in patients with 3 or 4 available H&E slides. NA: not available

The agreement in TILs scoring between different slides of the same patient as estimated by the ICC (two-way mixed effect model with single measurement) was .86 (95% CI .72-.94) which is interpreted as “good” to “excellent”.

**Supplementary table 10.** Average DCIS TILs score on H&E slides in relation to outcome

|  | **Median % TILs**  **all patients**  **(n=141)** | **Median % TILs cases (n=77)** | **Median % TILs controls (n=64)** | **Nominal *P*-value** | **FDR** |
| --- | --- | --- | --- | --- | --- |
| TILs (continuous)^a^ | 7.5 | 7.5 | 7.5 | 0.427 | 0.427 |
|  | | | | | |
|  | **DCIS patients (%)** | **Cases (n)** | **Controls (n)** | **Nominal *P*-value** | **FDR** |
| TILs category^b, c^  <1%  1-50%  ≥50% | 5 (3.5)  131 (93)  5 (3.5) | 4  68  5 | 1  63  0 | 0.054 | 0.216 |
| TILs category^b, c^  <1%  ≥1% | 5 (3.5)  136 (96.5) | 1  76 | 4  60 | 0.246 | 0.327 |
| TILs category^b^  <5%  ≥5% | 31 (22)  110 (78) | 14  63 | 17  47 | 0.160 | 0.321 |

Average TILs scores as scored by two breast pathologists on H&E slides of 141 DCIS patients treated with BCS alone. The interobserver agreement in TILs scoring as expressed by the intraclass correlation coefficient (ICC, two-way random effect model with average measurements and absolute agreement) was .95 (95% CI .92-.96) which is interpreted as “excellent”.

^a^: median of continuous TILs score. Comparisons between cases and controls were made with the Wilcoxon-Mann Whitney test.

^b^: TILs categories were compared between cases and controls with the Pearson Chi-squared test.

Nominal *P*-values were calculated with permutation. Multiple testing correction was performed using the FDR.

^c^: TILs scores are shown in predefined categorical groups.

**References**

1 Parra, E. R. *et al.* Immuno-profiling and cellular spatial analysis using five immune oncology multiplex immunofluorescence panels for paraffin tumor tissue. *Scientific reports* **11**, 8511, doi:10.1038/s41598-021-88156-0 (2021).

2 Parra, E. R. *et al.* Procedural Requirements and Recommendations for Multiplex Immunofluorescence Tyramide Signal Amplification Assays to Support Translational Oncology Studies. *Cancers (Basel)* **12**, doi:10.3390/cancers12020255 (2020).
